# Guide robots’ acceptance in organizations: user self-efficacy and robots’ organizational value

**DOI:** 10.3389/frobt.2026.1842941

**Published:** 2026-06-03

**Authors:** Kristel Marmor, Janika Leoste, Mati Heidmets

**Affiliations:** 1 Tallinn University of Technology, IT College, Tallinn, Estonia;; 2 Tallinn University, School of Educational Sciences, Tallinn, Estonia; 3 Tallinn University, Tallinn, Estonia

**Keywords:** emerging technologies, guide robots, self-efficacy, technology acceptance, technology adoption

## Abstract

**Introduction:**

This article explores the self-efficacy of guide robot (GR) users and assesses the value of integrating GRs into organizational workflows.

**Methods:**

The study consisted of two stages, both conducted in Estonia. First, we carried out a preliminary quantitative pilot study by applying the newly developed Guide Robot User Self-Efficacy Scale (GRUSES) in controlled and uncontrolled organizational settings. This pilot stage examined users’ confidence in using GRs across demographic variables, prior robot experience, and interaction contexts, and generated initial insights for the qualitative stage. In the main stage, we conducted semi-structured interviews with three stakeholder groups: GR end users, organizational administrators, and GR distributors.

**Results:**

The preliminary survey indicated that prior robot experience was associated with higher self-efficacy, whereas age and gender differences were not statistically significant in this sample. Users’ self-efficacy was lower in uncontrolled real-life use than in controlled guided scenarios, although this difference should be interpreted cautiously because the groups were not randomly assigned. The qualitative interviews, which form the core of the study, identified technical, user-related, and organizational barriers to integrating GRs into everyday workflows.

**Discussion:**

Based on these findings, we propose an exploratory three-actor framework linking robot capability, user readiness, and organizational readiness. The article also provides recommendations for guide-robot deployment in service organizations. As the GRUSES instrument remains under development, the survey results are interpreted as exploratory and hypothesis-generating.

## 1 Introduction

Recent advances in robotics and artificial intelligence (AI) have accelerated the development of social robots (SR) designed to operate alongside human personnel, providing various services of a social nature while interacting in meaningful ways with their users (Lee, 2021). According to (Darling, 2012), a social robot can be defined as “a physically embodied, autonomous agent that communicates and interacts with humans.” Human–Robot Interaction (HRI) in the context of social robots has been extensively studied (see e.g., (Arreghini et al., 2024)). A wide range of definitions for robotic technologies with social functionalities exist, and no universally accepted classification has been agreed upon. For example, (Feil-Seifer and Matarić, 2005), refer to categories such as assistive robotics, socially interactive robotics, and socially assistive robotics (the latter lying at the intersection of the first two). For the purposes of internal consistency, we consider Social Service Robots (SSR) as a particular type of SR. As (Wirtz et al., 2018) suggest, SSRs are “system-based autonomous and adaptable interfaces that communicate and provide services to the organization’s customers.” These robots are equipped with functionalities that allow them to learn from operations and adapt to new situations (Pagallo, 2013), recognize humans and other robots, and engage in social interaction (Latikka et al., 2019).

Adoption of AI-supported machine learning technologies in robotics has increased SSRs’ capabilities in HRI, enabling robots to engage in increasingly complex interactions and behave in a more human-like intelligent manner (Romero-C de Vaca et al., 2024; Leite et al., 2013). For example, humanoid robots such as Pepper and NAO have been used in educational settings to support learner engagement (Belpaeme et al., 2018), and the Care-O-bot has been deployed as a home assistant to help older adults and people with disabilities live independently (Asgharian et al., 2022; Bražinová et al., 2024). Because of these benefits, SSRs are being introduced in sectors that suffer from workforce shortages (e.g., healthcare, education, hospitality, and retail) where they provide services such as guidance, information delivery, companionship (Lee, 2021; Fasola et al., 2012), and virtual care (Turja et al., 2019). SSRs can increase organizational efficiency, raise productivity, improve client experience in service organizations, and help alleviate labour shortages (Lee, 2021; Wu et al., 2025; Mukherjee et al., 2021).

Despite successful implementation of robots in controlled environments (e.g., manufacturing), deploying robots in public and organizational spaces remains challenging (Zeller and Dwyer, 2022). Introducing robots into unstructured, real-world workflows often requires overcoming social, technical, and organizational obstacles (Lim et al., 2024). Key variables affecting the adoption of new technology include users’ attitudes (Savela et al., 2022), trust in technology (Pinto et al., 2022), and an individual’s confidence in their own ability to use the technology–i.e., user self-efficacy (Giger et al., 2025). Self-efficacy was defined by (Bandura, 1986) as an individual’s confidence in their ability to perform specific tasks in a specific environment. While SSRs hold great potential for numerous applications, their actual areas of use have so far remained narrow (Vishwakarma et al., 2024; Yoshimitsu et al., 2024). One reason may be the gap between controlled trials and real-world use: previous HRI research has primarily examined user attitudes (Savela et al., 2022), trust (Pinto et al., 2022), and perceptions (Arreghini et al., 2024) in laboratory settings or pilot projects, but there is limited understanding of how self-efficacy manifests in real organizational settings where robot interaction may be mandatory as part of one’s job (Zeller and Dwyer, 2022; Lim et al., 2024). This gap is significant because self-efficacy not only shapes individual user experiences but also influences organizational outcomes such as efficiency, employee satisfaction, and willingness to invest in robotics (Kong et al., 2025). By assessing user self-efficacy in both controlled and uncontrolled organizational contexts, the present study addresses these gaps and provides new insights into how confidence in robot use contributes to the successful adoption of guide robots in organizations.

Given the evidence that SSRs have greater potential than their current usage suggests, we undertook a two-part study in 2023–2024. First, we carried out an initial exploratory study focusing on robots’ nonverbal behavioural capacities. The results of this study indicated that a robot’s nonverbal behaviour expressed by gestures and expressions, is an important factor in improving users’ self-efficacy (Leos et al., 2024). Building on those results, in the present research we adopted a more systematic approach to examine additional factors that influence user satisfaction and organizational acceptance of robots, focusing on one type of SSR–the Guide Robot (GR). Person-following and guiding robots (GRs) are defined (Eirale et al., 2025) as having key application domains in healthcare and logistics (e.g., hospitals, warehouses, care facilities), as well as in enclosed public spaces such as museums, airports, and shopping malls. For our purposes, we define a *guide robot* as a subtype of SSR that performs user guidance tasks in socially structured service environments. In our study, a general-purpose robot platform (the TEMI v3 robot) was equipped with custom software to provide GR functionalities.

The present study does not aim to replace established technology-acceptance constructs such as perceived usefulness, perceived ease of use, trust, or attitudes. Instead, it treats user self-efficacy as a complementary explanatory construct. In TAM and UTAUT, perceived usefulness and perceived ease of use describe users’ evaluative beliefs about whether a technology improves task performance and whether it is effortful to use, while trust concerns expectations that the technology will behave reliably, safely, and predictably (Pinto et al., 2022; Davis, 1989; Venkatesh et al., 2003). Robot-related self-efficacy operates at a different but related level: it concerns whether users believe they can initiate, maintain, and recover an interaction with the robot in a situated service context. These constructs are therefore likely to interact. A guide robot that is perceived as useful, easy to use, and trustworthy can strengthen users’ self-efficacy because the interaction appears more understandable and controllable. Conversely, users with higher self-efficacy may be more willing to explore robot functions, tolerate minor breakdowns, and form more positive judgments of usefulness, ease of use, and trust during situated use (Latikka et al., 2019; Giger et al., 2025).

Such interaction is particularly important for guide robots because users often interact with them publicly, under time constraints, and in physical environments where navigation errors or unclear prompts are immediately visible. In such situations, acceptance depends not only on whether users regard the robot as useful or trustworthy, but also on whether they feel capable of acting competently with it. The link to organizational value follows from this cross-level mechanism. When users feel capable of using the GR, they are more likely to initiate interactions, complete guided tasks, seek assistance from the robot rather than from staff, and recover from minor interaction problems without abandoning the service. Repeated across many service encounters, these individual-level behaviours can contribute to smoother visitor flow, reduced staff interruptions, higher perceived service quality, and stronger organizational justification for continued investment. Accordingly, the contribution of this study is to connect robot-related self-efficacy with organizational value and to examine how this relationship changes when guide-robot use moves from controlled demonstrations to everyday organizational contexts.

To explore GR acceptance in organizations, we employed an exploratory sequential mixed-methods design. We first conducted a quantitative pilot study to gauge GR users’ self-efficacy under different conditions, and then a qualitative study as the main component to delve deeper into integration challenges and opportunities. Specifically, we addressed the following research questions (RQs):

- RQ1: How do GR users assess their self-efficacy, and does this assessment depend on their gender, age, and previous experience with robots?
- RQ2: Does users’ self-efficacy differ when using a GR in a controlled setting versus an uncontrolled real-life situation?
- RQ3: What are the expectations for GR features, and in what direction should GRs’ capabilities, skills, and qualities be developed to ensure better integration into an organization’s workflow?
- RQ4: What are the main barriers that currently limit GR users’ effectiveness in a workplace context, and what are the users’ behavioural expectations for a GR?
- RQ5: What are the potential application areas of GRs in organizations? What tasks could GRs perform, and what roles or jobs could be entrusted to them? How should organizations adjust to this new “workforce” of GRs?

In the following sections, we first review the conceptual background for GR acceptance, then describe our methodology, emphasizing the qualitative approach as the core study and the survey as a preliminary step. Next, we present the results, beginning with the qualitative findings and followed by the survey findings. We conclude with a discussion of the implications of these results and recommendations for both researchers and practitioners.

## 2 Conceptual model and previous studies

**FIGURE 1:**
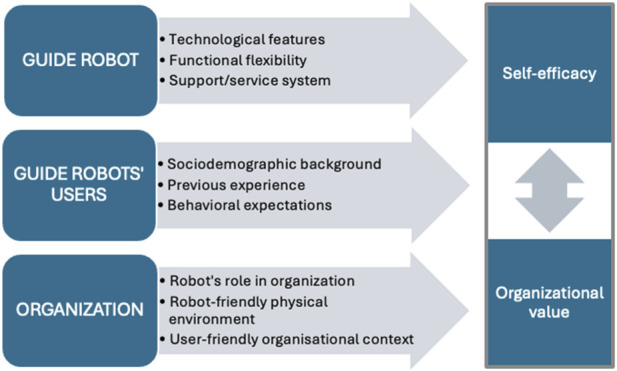
Conceptual model of Guide Robot acceptance in organizations.

To frame our study, we developed a conceptual model involving three key actors: the robot, the users, and the organizations deploying the robot. Unlike many prior studies that treat organizations as merely the context for HRI (Turja et al., 2020a; Bishop et al., 2019), we conceptualize organizations as active agents that invest in robotic technology and have distinct interests guiding their efforts. Our approach allows assessing GR acceptance from a dual perspective: (i) user self-efficacy (the user’s viewpoint) and (ii) organizational value (the organization’s viewpoint). By addressing both individual and organizational factors, this dual perspective allows a comprehensive analysis of GR acceptance. We outlined three main dimensions of influence for each actor (robot, user, organization), which determine their roles as either facilitators or barriers in the GR adoption process. Figure 1 presents the conceptual model of guide robot acceptance in organizations.

While much of the existing HRI research focuses on the interactions at the individual level (Bishop et al., 2019), being mostly carried out in controlled experimental settings, relatively few studies have been looking into these interactions within real-life organizational contexts (Zeller and Dwyer, 2022; Lim et al., 2024). In the latter case, additional difficulties emerge from the integration of robots in actual workflows. Additional complexities rise from the multi-stakeholder dynamics as well as from possibly diverging organizational goals. At the same time, further development and wider adoption of robots represent a growing opportunity for organizations to innovate and optimize their business processes (Latikka et al., 2019; Yoshimitsu et al., 2024).

Previous research has extensively addressed constructs such as trust, attitudes, and acceptance in HRI (Firmino de Souza et al., 2025). However, one of the less systematically examined factors in organizational contexts is user self-efficacy, despite its central role in Bandura’s social cognitive theory (Bandura, 1986; Bandura and Ramachandran, 1997; Bandura et al., 2006). For instance, (Aryati and Armanu, 2023), showed that self-efficacy positively influences employee commitment and ethical behaviour, while (Mustafa et al., 2019) found that organizational structure affects attitudes and behaviour, yet self-efficacy remains underexplored. Similarly, (Seikkula-Leino and Salomaa, 2020), framework highlighted the importance of self-efficacy in organizational development but emphasized the lack of systematic research in this area.

Bandura (Bandura, 1986; Bandura and Ramachandran, 1997; Bandura et al., 2006) emphasized that self-efficacy shapes how people approach challenges, the goals they set, and how persistent they remain when facing obstacles. In organizational HRI settings, employees and visitors with higher robot-related self-efficacy are more likely to interact effectively with robots, adapt to new workflows, and overcome technical or situational barriers. Conversely, low self-efficacy can amplify technophobia, stress, and resistance to change, which can reduce the organizational value of robots.

Although several self-efficacy scales related to robot use have been proposed (e.g., (Turja et al., 2020a; Robinson et al., 2020)), most of these have been validated in laboratory-like conditions or short-term interventions. These settings often lack the complexity of organizational environments where robot interaction may be mandatory and embedded into critical workflows. This represents a significant gap: the translation of self-efficacy from controlled to uncontrolled organizational contexts has not been systematically explored. Furthermore, organizations are still too often treated as passive contexts in HRI studies rather than as active stakeholders with distinct expectations and responsibilities in shaping successful adoption (Turja et al., 2020a; Bishop et al., 2019).

In this article, organizational value refers to the perceived contribution of guide robots to service quality, workflow efficiency, staff workload reduction, user satisfaction, and the organization’s capacity to innovate or maintain service continuity. We treat organizational value as a stakeholder-perceived construct rather than as a direct cost-benefit measure. The pairing of user self-efficacy and organizational value is therefore exploratory but theoretically grounded in a socio-technical understanding of technology adoption: guide robots can create value only when robot capability, user capability beliefs, and organizational support structures are aligned. A robot may be technically capable, but if its users do not feel being able to interact with it, or if the organization does not define its role and support routines, the technology may remain underused and fail to produce organizational benefit (Zeller and Dwyer, 2022; Bostrom and Heinen, 1977).

More specifically, our model treats the link between user self-efficacy and organizational value as a conditional cross-level pathway. At the individual level, self-efficacy shapes whether users approach the robot, follow its instructions, persist when prompts are ambiguous, and use recovery options after minor interaction breakdowns. At the interaction level, these behaviours affect task completion, the need for staff intervention, and the perceived smoothness of the service encounter. At the organizational level, the accumulation of successful interactions can be experienced as better service quality, improved workflow efficiency, reduced workload, and greater confidence in further robotics deployment. Low self-efficacy can interrupt this pathway by reducing use, increasing dependence on human staff, or making robot-related problems appear as personal failure. The mechanism is therefore not automatic: organizational value emerges only when individual capability beliefs are supported by reliable robot performance, role clarity, training, and a robot-friendly service environment (Zeller and Dwyer, 2022; Bostrom and Heinen, 1977).

To address these challenges, our study applies the proposed three-actor framework to identify factors that strengthen users’ self-efficacy and improve the organizational value of GRs. This approach contributes to filling two interrelated gaps: (i) the lack of empirical evidence on how self-efficacy differs between controlled and real organizational settings, and (ii) the limited treatment of organizations as active agents in HRI research. In doing so, we extend Bandura’s theoretical insights into the domain of guide robot adoption, illustrating how individual beliefs about capability and organizational readiness interact to determine successful integration.

Our conceptual model incorporates nine interrelated factors across the three domains of robots, users, and organizations. For GRs themselves, technological features such as navigation, sensors, and interaction capacities determine whether robots can reliably fulfil their guiding functions (Luo et al., 2024). Functional flexibility enhances adaptability across diverse contexts such as hospitals, airports, or museums, thereby increasing organizational value (Chu et al., 2019). A robust support and service system ensures long-term reliability, maintenance, and updates, which are prerequisites for sustained adoption (Cho and Kwon, 2025).

For users, sociodemographic background influences openness to or resistance against robots, with age, education, and cultural expectations shaping acceptance (Chu et al., 2019). Prior experience with technology strongly predicts user confidence, as positive experiences increase willingness to engage with robots while negative ones foster resistance (Chu et al., 2019). Users also bring behavioural expectations to the interaction; when robots meet these expectations through responsiveness, politeness, and smooth motion, interaction quality and satisfaction increase (Chen, 2018).

From the organizational perspective, three factors are critical. First, the robot’s role must be clearly defined to ensure effective workflow integration. Second, a robot-friendly physical environment (including space, accessibility, and charging infrastructure) is essential. Finally, a supportive organizational culture that provides training and fosters innovation ensures that both employees and visitors feel confident and motivated to use robots (Cho and Kwon, 2025).

The nine factors depicted in Figure 1 are not intended as nine independently validated predictors. Instead, they are organized into three higher-order socio-technical constructs. First, robot capability includes technological features, functional flexibility, and support/service systems. Second, user readiness includes sociodemographic background, previous experience, and behavioural expectations. Third, organizational readiness includes the robot’s role in the workflow, the robot-friendliness of the physical environment, and the user-friendliness of the organizational context. This structure allows the model to move beyond a descriptive list by showing that acceptance depends on alignment among technical capability, user capability beliefs, and organizational support. The model is therefore proposed as an exploratory framework for guide-robot deployment and should be operationalized and tested in future studies rather than interpreted as a validated predictive model.

Our study advances the understanding of GR acceptance by introducing a comprehensive, three-actor framework that simultaneously considers technological, psychological, and organizational factors. By integrating user self-efficacy and organizational value into one model, it bridges individual and institutional perspectives and highlights the interdependence between user confidence, robot capability, and organizational support.

### 2.1 Social service robots

Recent advancements in AI and machine learning have significantly enhanced SSR capabilities, making them viable in a variety of organizational settings. These include hospitals, universities, libraries, tourist attractions, theme parks, shopping malls, and museums (Adetayo et al., 2023; Iio et al., 2020; Ogle et al., 2019; Shahzad et al., 2024; Gasteiger et al., 2021; Maniscalco et al., 2024). Contemporary SSRs are equipped with sophisticated navigation systems, interactive interfaces, and other functionalities that allow them to become increasingly context-aware and to offer seamless, personalized assistance to users. The future of SSRs appears promising as these technologies continue to evolve, increasing SSRs’ usability for different tasks in a variety of environments (QViro, 2024).

Our study focuses on GRs - SSRs configured to assist users in navigating and finding their way in public spaces. The concept of the guide robot dates to the 1970s, when projects such as MELDOG were developed in 1976 to help visually impaired individuals navigate their environment (Tachi et al., 1985). Over the decades, GRs have been utilized in various settings. For example, tour guide robots have been deployed in museums to provide interactive guided tours, and in healthcare facilities to alleviate shortages of guide dogs and human guides (Thrun et al., 2000; Degeurin, 2024).

Despite this growth in SSRs capabilities and applications, including the functionalities that allow them being used in the capacity of GRs, there are still barriers that limit their broader adoption and use, including technical limitations, lack of functional flexibility, and inadequate support and service systems.

*Technical barriers* are among the most evident challenges to SSR adoption and directly influence HRI and user self-efficacy. Common technical issues include system malfunctions (software or hardware failures) that can be critical in dynamic, unpredictable public spaces. GRs in particular may struggle with navigation in complex environments featuring obstacles such as narrow corridors, uneven flooring, or crowded areas (Mosquera-Maturana et al., 2025; Eirale et al., 2025; Rivero-Ortega et al., 2023). Non-intuitive user interfaces can exacerbate these challenges for people lacking experience with advanced technology, creating accessibility problems and hindering effective HRI (Ogle et al., 2019).

Another barrier to GR adoption is *limited functional flexibility*. Many robots currently available are designed for specific purposes and cannot be easily repurposed for different environments. For example, a robot designed for museum guidance is not immediately suitable for hospital work because it would require different navigation patterns and interaction protocols. To improve flexibility, SSRs need to be customizable for specific tasks and capable of seamlessly navigating complex, uncontrolled environments. Enhancing these abilities would allow social robots to assume different roles across settings, broadening their range of use (Frieske et al., 2024).

*A lack of robust support and service systems* is also a critical barrier to robot adoption. At present, many distributors and vendors cannot offer comprehensive maintenance, repair, and customer support services–essential components for any organization integrating SSRs into their workflows. Without effective support, organizations face operational risks that can diminish confidence in the technology. Addressing this challenge requires structured after-sales support infrastructure, including regular maintenance services, troubleshooting protocols, and training programs for both end-users and support staff (Sadangharn, 2022; Chen, 2018). Improved collaboration between manufacturers, distributors, and end-users is needed to ensure SSRs remain functional and are aligned with the specific needs of their deployment environments (García et al., 2020).

### 2.2 Users of social service robots

The typical SSR user is someone who relies on a robot to perform a specific task–for example, navigating to a destination, obtaining information about an exhibit, or scheduling activities in a transit hub (Iio et al., 2020). Data suggest that SSR use is more common among younger individuals, urban residents, and professionals in service sectors, pointing to demographic patterns in robot adoption (Carradore, 2022; González-Anleo et al., 2024). Prior experience with robots is an important factor shaping user interactions - familiarity tends to increase confidence and reduce apprehension (Savela et al., 2022).

User-related issues are among the most complex factors affecting SSR adoption and self-efficacy. These challenges stem from individual perceptions, behaviours, and attitudes toward technology. Unfamiliarity with robots can hinder acceptance and willingness to interact, whereas prior knowledge of robots often leads to greater acceptance (Maj et al., 2023). Job security concerns can also create resistance if employees perceive robots as threats to their roles (Turja et al., 2019). Trust in the robot’s reliability is critical: users are less likely to engage with robots they perceive as prone to errors or ineffective at tasks.

Users have also behavioural expectations for robots that influence acceptance. People expect robots to follow certain social and communication norms–for instance, respecting personal space (proxemics), using appropriate gaze and gestures, and generally behaving in a socially acceptable manner (Yi and Park, 2024). Privacy concerns are increasingly relevant as robots collect, store, and process user data. Transparent policies and strong ethical guidelines on data security and respect for user autonomy and dignity are essential for building trust (Vincent et al., 2015).

*Nonverbal behaviour* is emerging as a critical component of HRI, with growing recognition of its importance in strengthening user engagement, trust, and self-efficacy (Saunderson and Nejat, 2019). Gestures, facial expressions, eye contact, proxemics, and other nonverbal cues significantly improve a robot’s perceived social presence and make interactions feel more natural (Spatola et al., 2021). Takayama and Pantofaru (Takayama and Pantofaru, 2009) observed that people are more likely to engage with robots that exhibit human-like proxemic behaviour. A robot’s expressions also aid in interpreting its responses and improving coordination–for example, even minimal gaze cues and responsive body language increase a robot’s perceived attentiveness (Rifinski et al., 2020). Accompanying verbal instructions with appropriate body and hand gestures enhances communication, making messages clearer and more engaging (Bremner and Leonards, 2016). A robot’s expressiveness, for example, richer facial and bodily expressions, can evoke stronger empathetic responses, allowing users to relate emotionally (Konijn and Hoorn, 2020).

Previous studies indicate that appropriate nonverbal cues increase user satisfaction and their confidence in robots (Li et al., 2022). For example, animated facial expressions, smooth movements, and responsive behaviours help users feel more at ease, thereby increasing their willingness to engage with the robot. Also, nonverbal behaviour adjusted according to specific cultural norms and social expectations can further contribute to building trust and comfort, making the interaction more intuitive and natural (Leos et al., 2024; Baxter et al., 2016).

### 2.3 Organizational context

Organizations are increasingly looking to integrate SSRs into their workflows, motivated by potential gains in efficiency, improved customer service, and an innovative public image (Almokdad and Lee, 2024). SSRs can take on different roles in organizations, often handling routine, repetitive tasks with consistent efficiency and accuracy. These tasks–such as guiding visitors, providing information, or managing simple logistics–can streamline operations and free human staff to focus on more complex responsibilities (Turja et al., 2019; Shahzad et al., 2024; Adetayo et al., 2023). For example, in libraries and hospitals SSRs might reduce administrative burdens while enhancing user experience through intuitive navigation assistance and personalized interactions (Leos et al., 2024; Leoste et al., 2024; Jadad-Garcia and Jadad, 2024; Turja et al., 2019). Beyond functional benefits, integrating robots can signal an innovative and forward-thinking organizational culture (Tanase, 2023; Blaurock et al., 2022).

However, achieving the full potential of SSRs requires overcoming significant organizational barriers. Organizational readiness is critical, including both physical infrastructure and human factors. Infrastructure must often be adjusted to be robot friendly. Physical spaces may need modifications–e.g., wider pathways, ramps instead of stairs, clear signage–to facilitate robot navigation and operation. Environmental features like narrow corridors, door thresholds, stairs, uneven floors, or crowded areas can hinder robots (Gross et al., 2017; Moro et al., 2019; Mosquera-Maturana et al., 2025). Ensuring a robot-friendly physical environment can pose challenges and entail upfront costs.

Equally important is creating a user-friendly organizational context for the introduction of robots (Tan et al., 2016). This includes educating visitors or customers about the robot’s functionalities and role, which helps set appropriate expectations and reduce anxiety. It also involves training employees so that staff are prepared to interact with, manage, and maintain the robots. Clearly defining the workflow integration of SSRs is necessary–organizations must specify the robot’s role and how human workers should collaborate with it. Robust support systems should be in place, such as maintenance routines and emergency protocols for robot malfunctions or user safety concerns. A proactive approach–actively seeking user and staff feedback and refining processes–can greatly enhance integration (Turja et al., 2020b).

Adapting SSRs to diverse tasks and cultural contexts is another organizational consideration. Robots may need modular design and AI-driven personalization to meet specific needs: assisting older patients in hospitals, guiding multilingual international visitors in museums, etc. When robots display adaptive behaviour and tailored nonverbal communication, user trust and satisfaction tend to grow (Saunderson and Nejat, 2019). Effective use of nonverbal cues like proper distancing and expressive “body language” contributes to more natural human-robot interactions (Takayama and Pantofaru, 2009). Ultimately, the value SSRs bring goes beyond performing tasks–when properly integrated into workflows and aligned with user expectations, they can help transform service models to be more adaptive, efficient, and user-centric.

### 2.4 User self-efficacy

As robots become increasingly integrated into social environments, it is crucial to assess their design and social impact to achieve productive HRI (Ang et al., 2024). Various criteria have been proposed as bases for such assessments–for instance: anthropomorphism, behavioural realism, sociability, warmth, competence, emotional acceptance, agency, human-likeness, communication quality, and nonverbal behaviour (Mandl et al., 2022; Spatola et al., 2021; Leoste et al., 2024). Other important aspects include attitudes toward technology (Savela et al., 2022), trust in technology in specific contexts (Pinto et al., 2022), and individuals’ confidence in their ability to control robotic technologies (Rosenthal-von der Pütten and Bock, 2018), or, in other words, user self-efficacy (Bandura, 1977).

An increasing number of studies are using robot users’ self-efficacy as a criterion for assessing readiness to accept robots, e.g., (Lin et al., 2025; Liao et al., 2023). Self-efficacy, defined as an individual’s belief in their ability to perform specific tasks in a specific environment (Bandura, 1986), is particularly important for various SSR contexts, where users must feel confident in their ability to interact effectively with robots (Robinson et al., 2020). Unless people believe they can produce desired effects by their actions, they have little incentive to act or to persevere in the face of difficulties (Bandura, 2018). High self-efficacy can lead to greater engagement and persistence, resulting in better performance and satisfaction (Heslin et al., 2006).

In the context of HRI, users with higher self-efficacy are more likely to interact effectively with robots, adapt more quickly to new technologies, and use robots more productively for their tasks. Self-efficacy thus shapes not only users’ initial willingness to engage with a robot, but also their long-term adoption, satisfaction, and overall experience with the technology. As (Li et al., 2022) note, *“Self-efficacy helps determine how much effort individuals expend on an activity, how long they persevere when confronting obstacles, and how resilient they are in the face of unfavourable situations.”* This applies to HRI by influencing both practical and emotional dimensions of interaction. When users feel efficacious, they are more likely to trust the robot, engage with it, and view it as a helpful tool rather than a source of frustration or threat. Higher self-efficacy can mitigate technophobia and anxiety, highlighting the importance of training programs and design choices that build user confidence (Bandura, 1986).

Prior research provides evidence of self-efficacy’s role in HRI. For instance (Robinson et al., 2020), developed a robot self-efficacy scale and found that interactions with a robot instructor increased participants’ self-efficacy, agreeableness, and willingness to interact with robots. The influence of the nonverbal behaviour of social robots on user perceptions and self-efficacy was examined by Leos et al. (2024), Leoste et al. (2024), finding that appropriate nonverbal cues can indeed increase user trust and satisfaction. Additionally, studies by (Belpaeme et al., 2018; Kennedy et al., 2017; Mendoza et al., 2024) demonstrate the importance of social feedback and cultural context in HRI across different scenarios.

In our study, we chose to use user self-efficacy as a key indicator for evaluating not only the users’ ability to effectively perform tasks with a GR, but also their comfort and confidence during such interactions. By focusing on self-efficacy, we aimed to capture a holistic picture of users’ competence, resilience, and satisfaction when using GRs.

## 3 Methodology

### 3.1 Research design overview

To meet our objectives of identifying factors that improve users’ self-efficacy and increase the organizational value of GRs, we employed a mixed-methods sequential exploratory design. We conceptualized GR acceptance as involving three active agents - the robot, the users, and the organization, and assessed it from both user and organizational perspectives, as described in our conceptual model. Our empirical study was carried out in two stages. First, we conducted a quantitative pilot experiment to measure guide robot users’ self-efficacy in two different settings (controlled vs. uncontrolled environments) and across different user characteristics. This initial stage was intended to explore baseline patterns and inform the subsequent inquiry. We then followed up with a qualitative study involving focus-group interviews with key stakeholders, which served as the main study to delve into the barriers, expectations, and opportunities for GR integration in organizational workflows.

The two empirical stages were connected sequentially. The survey stage was used as an exploratory sensitizing step rather than as a standalone validation study. Its findings indicated that prior robot experience mattered for self-efficacy and that self-efficacy was lower in real-life use than in controlled guided use. These findings informed the qualitative interview protocol by directing attention to three issues: how users interpret robot behaviour in less supported situations, what kinds of training or support could strengthen users’ confidence, and what organizational arrangements are needed when a guide robot becomes part of a workflow rather than a demonstration object. The qualitative stage then examined these issues from the perspectives of end-users, administrators, and distributors, enabling interpretation of the survey patterns through stakeholder experiences. The adopted sequential approach allowed the findings from the preliminary survey to guide the topics and questions in the interviews. All research activities took place in Estonia and were approved by the relevant institutional review processes. Below, we describe the methodology of each stage in detail.

### 3.2 Participants, robot, and setting

**FIGURE 2:**
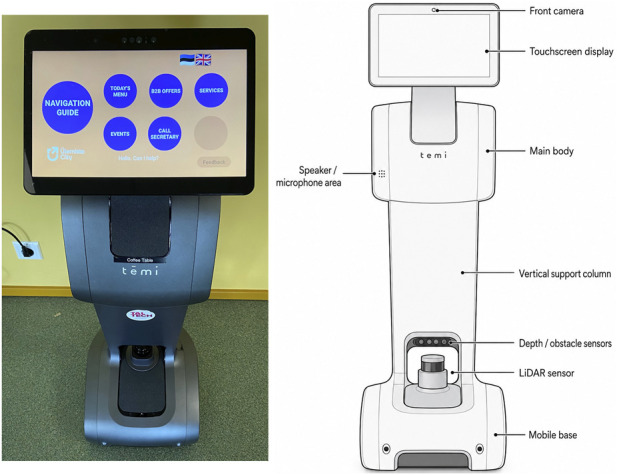
TEMI v3 robot’s GR interface (left) and the robot’s main components (right).

Throughout both stages, we utilized a semi-autonomous social service robot, TEMI v3, configured with guide-robot functionalities (see Figure 2 for the robot’s interface and main components). TEMI is an AI-assisted mobile robot equipped with features such as autonomous navigation, pre-mapped location guidance, and human-robot communication capabilities. It can display expressive facial animations on its screen, perform responsive movements, and adhere to proxemic norms (Leos et al., 2024; Leoste et al., 2024). To unlock TEMI’s full capabilities (e.g., room mapping, smartphone integration, video calls), we used the TEMI Center Pro service. We also developed custom software for TEMI to enable specific GR behaviours needed for our study. All experiments and interactions took place on the premises of host organizations (e.g., office building, university library, hospital), providing realism while maintaining a controlled scope.

### 3.3 Study I: interviews with key stakeholders

#### 3.3.1 Research questions

The qualitative stage of the study aimed to identify practical barriers and enablers for integrating guide robots into organizations’ everyday workflows. We were particularly interested in understanding stakeholders’ experiences and expectations regarding GR behaviour and how organizations might need to adapt to facilitate GR adoption. In line with RQ3, RQ4, and RQ5 (the research questions assigned to this stage), this portion of the research sought to answer: What improvements in GR features and capabilities do users and managers expect for better workflow integration? What are the main barriers currently hindering effective GR use, and what are users’ behavioural expectations of GRs? And finally, what potential roles and tasks could GRs take on in organizations, and how should organizations adjust to effectively incorporate GRs as part of their workforce?

#### 3.3.2 Interview instrument

We conducted semi-structured focus group interviews with representatives of three participant groups: (a) End-users of GRs–employees (e.g., library staff, hospital and care facility staff, office workers): who had directly interacted with a guide robot in their workplace; (b) Organizational administrators or managers–individuals responsible for implementing or overseeing the use of robots in their organization; and (c) Distributors/technical support personnel–representatives of companies that sell or rent GRs, who have in-depth knowledge of the robot’s functionalities and post-sale support challenges. All interview participants had prior experience with a GR deployed in an organizational setting, where a robot (the TEMI v3) had been used as an in-house guide for at least 1 week.

The interview protocol was designed to explore the role, user-friendliness, and development needs of GRs within organizations. For end-users and administrators, questions prompted participants to reflect on tasks they found GRs suited or not suited for, and to envision future roles for the robot in their organization. Discussions covered how easy or appropriate the robot was for various employees and visitors to use, any concerns users had with current GR models (e.g., technophobia, privacy issues), and suggestions for an “ideal” guide robot that would improve effectiveness and user satisfaction. Participants were asked to describe what an ideal GR would look or behave like, compared to current models, to pinpoint desired improvements.

A second set of questions focused on users’ behavioural expectations of the robot, especially regarding the robot’s non-verbal behaviour. We asked how aspects such as the robot’s body language, movement speed, interaction distance, facial expressions, and gestures influenced participants’ confidence and trust. Participants were encouraged to discuss any specific robot behaviours that made them feel comfortable. They also suggested improvements in these behavioural patterns that could strengthen user confidence and cooperation. For instance, we probed which non-verbal cues or interaction styles participants felt would make the robot more “friendly,” intuitive, or trustworthy during use.

In the interviews with distributors, we focused on post-deployment experiences and broader adoption factors. We asked about how communication with client organizations occurs after a sale or deployment: What training is provided to new users? What common questions or problems do customers report? What support is available to integrate the GR into specific tasks or environments? Distributors also offered their perspective on how to increase GR adoption in the market–for example, what design improvements or added functionalities are most often requested by customers, and what features might make the robots more understandable and valuable to organizations.

The interview protocol was refined in light of the preliminary survey findings. Because the survey suggested that prior robot experience was associated with higher self-efficacy, participants were asked about training, first encounters with the robot, and the kinds of guidance that would make novice users more confident. Because the survey also suggested lower self-efficacy in uncontrolled real-life settings, the interviews included questions about situations in which users felt uncertain, unsupported, observed by others, or unsure how to recover from interaction problems. These questions later informed the thematic coding of technical reliability, training and support, robot role clarity, proxemics, gaze, and privacy as barriers or facilitators of guide-robot acceptance.

#### 3.3.3 Sample

We conducted semi-structured focus group interviews with representatives of three participant groups: (a) end-users of GRs–employees (e.g., library staff, hospital and care facility staff, office workers): who had directly interacted with a guide robot in their workplace; (b) organizational administrators or managers–individuals responsible for implementing or overseeing the use of robots in their organization; and (c) distributors/technical support personnel–representatives of companies that sell or rent GRs, who have in-depth knowledge of the robot’s functionalities and post-sale support challenges. All interview participants had prior experience with a GR deployed in an organizational setting, where a robot (the TEMI v3) had been used as an in-house guide for at least 1 week.

A total of 26 individuals participated in the qualitative interviews, drawn from various organizations that had experience using a social service robot as a guide. This sample included representatives from healthcare (a university hospital and a nursing home), academia (a technological university’s IT College and academic library), a smart city office environment (Ülemiste City business campus), and robotics retail companies (e.g., Roborent OÜ, Kinema OÜ, PureSec GmbH). Each focus group was composed of participants with similar roles (e.g., end-users together, managers together, distributors in their own group) to encourage open discussion of shared experiences.

#### 3.3.4 Procedure

The interviews were conducted between June and December 2024. Participants were invited via email with an explanation of the interview’s purpose and how the collected data would be used. At the start of each interview, we obtained verbal consent (or written consent, for two participants who opted to give answers in writing) and assured participants of confidentiality. Each focus group session lasted approximately 35–45 min. Most interviews took place in person at a location convenient to the participants; a few were conducted via video conferencing due to availability or distance. With consent, interviews were audio-recorded for accuracy (except for two written-response cases). We used an open-ended interview protocol that allowed participants to freely share experiences, perceptions, and suggestions related to guide robots. Interviewers followed a predefined set of themes aligned with our RQs but also encouraged participants to bring up any relevant issues from their own perspective. After the recorded session, the audio was transcribed. In transcription, we paraphrased where necessary to remove any personally identifying details while preserving the meaning of responses.

#### 3.3.5 Data analysis

We analysed the interview data using thematic analysis (Braun and Clarke, 2006) to systematically identify and interpret patterns in participants’ responses. The thematic analysis followed a consensus-based procedure. Two researchers first read the transcripts independently and generated preliminary codes. They then compared the code lists, discussed differences in interpretation, and produced a shared coding structure. The final coding was therefore not the result of a purely independent coding procedure but of negotiated consensus. For this reason, Cohen’s kappa was not calculated.

Next, they generated initial codes by highlighting segments of text that captured important concepts or issues raised by participants. These codes were descriptive labels (e.g., “navigation issues,” “privacy concern,” “needs training”) corresponding to recurring ideas in the data. The two coders then compared and refined their codes through discussion, merging similar codes and resolving any discrepancies to ensure consistency.

After coding, we organized the codes into broader themes. Related codes were grouped under candidate themes and sub-themes, which were iteratively reviewed against the data to ensure they accurately reflected participants’ views. The coding and theme development process was collaborative: regular meetings between the coders were held to reach consensus on themes and to verify that interpretations were grounded in actual participant quotes. This collaborative approach enhanced the reliability of our qualitative analysis. Ultimately, we identified three overarching thematic categories of barriers and improvement areas: Technical Capabilities, Users’ Expectations, and Organizational Arrangements. Within each category, several specific sub-themes were defined (see Table 1 for an overview).

- *Technical Capabilities:* This category focuses on challenges posed by the current design and functionality of GRs. Sub-themes here included Technical Reliability (ensuring the robot operates without malfunctions), Smooth Navigation (the ability to move effectively through various physical environments), Language/Communication Interface (providing a user-friendly, multilingual interaction interface), and Customization to Tasks (the ability to adapt GR functionalities to specific tasks and user needs). These issues represent technical barriers that can hinder GR performance and user confidence.
- *Users’ Expectations:* This category captures barriers stemming from user perceptions, needs, and comfort levels. Sub-themes included Privacy (ensuring GR interactions do not infringe on personal data or make users feel observed), Proxemics (maintaining appropriate distance and positioning during interactions), and Gaze (using eye contact or facing users in a way that feels respectful). These aspects reflect what users expect from a robot’s social behaviour and how deviations can cause discomfort or distrust.
- *Organizational Arrangements:* Barriers in this category arise from the interplay between GRs and workplace dynamics or processes. Sub-themes included GR’s Role in Organizational Workflow (the importance of clearly defining what the robot should do and its responsibilities in processes), Customized Infrastructure (adjusting the physical and IT infrastructure to accommodate GR operations, such as space for the robot to move and integration with organizational systems), and Training and Support (providing adequate training to staff and ongoing support to ensure effective use of the robot). These themes highlight what the organization can do to facilitate or impede successful GR integration.

**TABLE 1:** Summary of the identified themes and sub-themes with descriptions.

Category	Sub-theme	Description
Technical capabilities	Technical reliability	Ensuring reliable, malfunction-free operation of GRs
	Smooth navigation	Facilitating effective movement through various physical environments
	Language/Communication interface	Providing user-friendly interaction in the preferred language and intuitive interface
	Customization to tasks	Adapting GR functionalities to specific tasks and user needs
User’s expectations	Privacy	Ensuring that GR interactions respect personal data and intimacy
	Proxemics	Maintaining appropriate spatial distance and positioning in user interactions
	Gaze	Orienting visual sensors respectfully, making appropriate eye contact
Organizational arrangements	GR role in organizational workflow	Defining clear roles and responsibilities for GRs within workplace processes
	Customized infrastructure	Adjusting the physical setting to accommodate GR operations (e.g., space, signage)
	Training and support	Offering adequate preparation, instruction, and ongoing assistance to users and staff

### 3.4 Study II: developing robot user’s self-efficacy scale

#### 3.4.1 Research questions

In the first, exploratory stage of our research, we focused on assessing users’ self-efficacy in operating a guide robot, with attention to basic user characteristics and context effects. This stage addressed RQ1 and RQ2: we asked how users evaluate their own ability to use a GR and whether this evaluation varies by gender, age, or previous experience with robots (RQ1), and whether the context of use–a controlled experimental scenario versus an uncontrolled real-life scenario–affects users’ self-efficacy (RQ2).

#### 3.4.2 Research instrument

Various instruments exist to assess perceptions of robots and HRI, such as the Godspeed Questionnaire (Bartneck et al., 2009) and the Robot Social Attribute Scale (RoSAS) (Ca et al., 2017). The Human-Robot Interaction Evaluation Scale (HRIES) (Spatola et al., 2021) measures dimensions like sociability (e.g., “Warm,” “Friendly”), animacy (e.g., “Natural,” “Human-like”), agency (e.g., “Intelligent,” “Intentional”), and disturbance (e.g., “Creepy,” “Weird”). Other recent scales include Social Perception of Robots Scale (SPRS) (Mandl et al., 2022) with dimensions of sociability, competence, morality, and anthropomorphism. While these scales gauge general perceptions of robots, our focus was specifically on user self-efficacy in using a guide robot. Some prior instruments measure robot-related self-efficacy (e.g., the RUSH-3 scale (Turja et al., 2020a; Pinto et al., 2022; Rosenthal-von der Pütten and Bock, 2018)), but those were designed largely for laboratory settings or short-term interactions and do not directly apply to GR use in public organizational spaces. Therefore, we constructed a new scale tailored to this context, the Guide Robot User Self-Efficacy Scale (GRUSES), to capture how confident users feel using a GR in an indoor public area of an organization. In developing GRUSES, we followed Bandura’s (Bandura et al., 2006) guidelines for creating self-efficacy measures. The scale was constructed following Bandura’s recommendation that self-efficacy measures should be task- and context-specific. Bandura advises that self-efficacy scales be context-specific rather than one-size-fits-all, that items should accurately reflect the construct (self-efficacy being a judgment of capability to perform certain actions), and that the scale should be empirically validated through piloting and refinement. Applying these principles, we went through three steps to create and refine GRUSES:

1. Expert panel brainstorming: Three experts in HRI and psychology generated an initial pool of 17 items intended to assess a person’s sense of efficacy while using a guide robot. Based on theoretical considerations, the panel anticipated two underlying dimensions: *operational efficacy* (perceived difficulty, clarity, and effectiveness of using the GR for tasks) and *affective efficacy* (the perceived comfort, pleasantness, and sense of safety when interacting with the robot).
2. Pilot testing and refinement: We conducted a pilot survey with as small samples (N = 28). Participants in the pilot trial used the GR in a sample scenario and then answered the draft questionnaire. Based on their feedback and response patterns, we reworded items that were ambiguous or confusing. We also found one important aspect was missing and added an item to cover it. This process resulted in a refined set of items.
3. Survey study: The revised scale, now with 12 items, was administered in the present study’s survey (with N = 226 participants; see below for sample details). In the Results section, we report descriptive statistics and evaluate the scale’s reliability and validity based on this data.

The final GRUSES instrument (see Supplementary Appendix 1 for the full item list) includes statements that respondents rate on a 7-point Likert scale (1 = “Strongly disagree” to 7 = “Strongly agree”). Example items include assessments of how difficult or easy it was to communicate with the robot, how well the user understood the robot’s messages, how secure they felt interacting with it, the robot’s reliability as a partner, the pleasantness of the interaction, the user’s self-confidence during use, perceived effectiveness and speed of task completion with the robot, and the naturalness of working with the robot. We also collected demographic information (gender, age group) and data on participants’ previous experience with using robots, to analyse subgroup differences. GRUSES should be interpreted as an exploratory, context-specific measure developed for the present guide-robot setting.

#### 3.4.3 Study design

**FIGURE 3:**
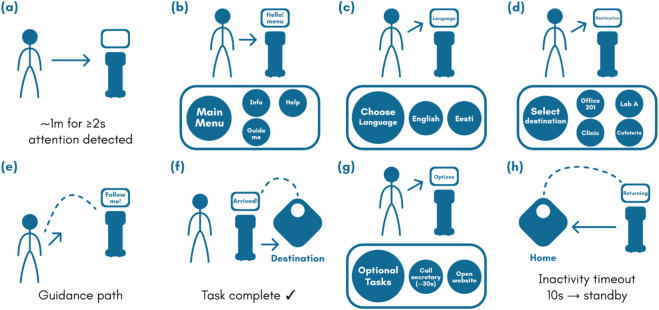
Procedure of the experiments. **(a)** User attention is detected when the participant approaches the robot, approximately 1 m for at least 2 s. **(b)** The robot greets the user and displays the main menu. **(c)** The user selects the communication language. **(d)** The user selects the desgnation. **(e)** The robot guides the user along the route. **(f)** The robot announces arrival and task completion. **(g)** Optional follow-up tasks are displayed, including calling a secretary or opening a website. **(h)** After an inactivity timeout of approximately 10 s, the robot returns to its home position and standby mode.

We assessed GR users’ self-efficacy using the TEMI v3 robot (as depicted in Figure 3) in two distinct settings: a controlled environment and an uncontrolled (real-life) environment. This comparison was designed to reveal any differences in user experience and confidence between a scenario where support and guidance are provided versus one where users must interact with the robot autonomously. Importantly, both the controlled and uncontrolled trials were conducted in authentic public spaces within host organizations (rather than in a laboratory), to increase ecological validity while still distinguishing the level of guidance provided.

- Controlled setting: In the controlled scenario, participants were asked to perform a series of tasks with the robot under the supervision of researchers. We prepared a predefined usage scenario and gave participants step-by-step instructions on how to interact with the robot to complete the tasks. Specifically, participants were told they would use the robot to navigate to various locations and perform simple actions at those locations. The scenario proceeded as follows: the participant approaches the robot and selects a communication language on the touchscreen; they choose a destination from the menu (e.g., a certain office or room); the robot then leads the participant to that destination. Upon arrival, the participant is prompted (by the scenario script) to either place a video call to a “secretary” using the robot’s interface or to open the organization’s website on the robot’s screen (simulating information retrieval). After completing these actions, the participant follows the robot to a final location (e.g., a lobby or relaxation area) to conclude the session. Throughout this controlled session, the researcher stayed nearby, offering guidance or clarifications if the participant seemed confused. The key characteristics of the controlled setting were that participants knew they could rely on help if needed and that using the robot was voluntary and meant to be a “safe” trial–they could always opt out or ask for assistance. This supportive environment was intended to reduce stress and encourage participants to explore the robot’s features.
- Uncontrolled setting: In the uncontrolled scenario, the guide robot was deployed as it would be in a real public setting, and participants used it without direct researcher assistance. The robot stood in a public area (for example, an office building lobby or library entrance) and would activate its greeting when someone came within its sensor range. Participants in this condition were asked to approach the robot as if they were regular users or visitors, and to utilize it to reach a destination of their choice. The robot itself initiated interaction by greeting the user with an animated face on its screen and bringing up the user menu. Participants independently navigated the interface–selecting their language and desired destination from the options. Once they made a selection, the robot gave spoken instructions and autonomously guided the participant to the chosen location. Upon reaching the destination, the robot announced task completion, bid the participant goodbye, and then returned on its own to its start position to await the next user. In this mode, the additional actions (calling the secretary or viewing the website) were available but not explicitly prompted, to mirror a more natural usage scenario (the participant could choose whether to make a call or get information). The uncontrolled scenario effectively required participants to rely entirely on the robot and their own ability to use it, as if the robot were a permanent service with no human assistant present. After finishing the interaction, participants were approached by the researcher and asked to fill out the feedback questionnaire (GRUSES) if they were willing and had time. The researchers observed quietly from a distance during the interaction to note any issues but did not intervene.

##### 3.4.3.1 Key differences from the user’s perspective

The core task (having the robot guide the participant to a location) was the same in both conditions, to allow comparison. However, the *level of guidance and autonomy* was the main difference. In the controlled environment, participants had the safety net of researcher support and knew that using the robot was optional (“for fun” or practice). In the uncontrolled environment, participants were effectively “on their own” with the robot, and the situation simulated a real workflow where using the robot might be necessary to accomplish the task. We anticipated that these differences could affect participants’ confidence and stress levels: the controlled condition might make them feel more supported and thus more confident, whereas the uncontrolled condition might reveal challenges that reduce self-efficacy. This dual-context approach allowed us to examine how context influences user self-efficacy, trust, and overall experience with the GR.

#### 3.4.4 Procedure

The pilot experiments were conducted from December 2023 to May 2024 across six different public venues in Estonia. We selected venues relevant to GR use, including: an office building lobby (Ülemiste City business campus), a university administrative floor (the IT College at Tallinn University of Technology), a technology fair (sTARTUp Day in Tartu), a tech conference venue (TalTech Mektory center), a public industry conference (Industry 5.0 conference in Tartu), and a university library (the Academic Library of TalTech). The controlled/uncontrolled comparison should be interpreted as quasi-experimental, as the participants were not randomly assigned to conditions, and the controlled and uncontrolled trials differed in timing and venue. In total, five controlled-environment trials were run between December 2023 and February 2024 (one at each of five venues), and two additional trials in uncontrolled environments took place in May–June 2024 (at the Ülemiste City lobby and the TalTech library).

**FIGURE 4:**
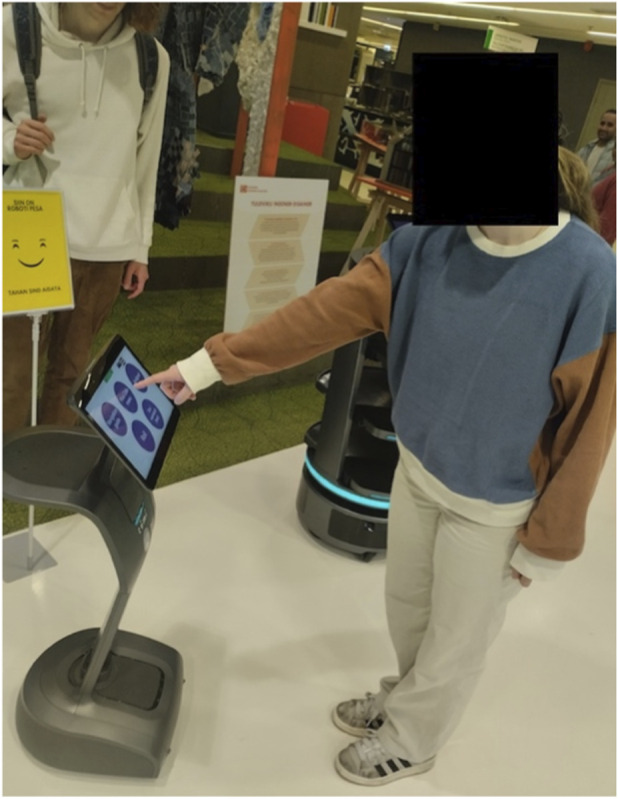
Experimental setup: A snapshot of a participant interacting with the TEMI robot during the controlled experiment, illustrating the user following the robot’s guidance.

Each participant in the study went through the following steps, tailored to whether they were in a controlled or uncontrolled trial:

- Step 1: Preparation (controlled trials only). Before a controlled experiment began, a researcher met with the participant individually and provided detailed instructions about what would happen. The participant was briefed on the sequence of actions they would perform with the robot (as outlined in the scenario above). For example, the researcher explained: “You will approach the robot, choose the language and a destination, follow the robot, and when you arrive you will use the robot to call a secretary and also open a webpage, then proceed to a final location.” Once the participant indicated they understood and felt ready, the interaction commenced with the participant approaching the robot positioned at its start point in the corridor or lobby.
- Step 2: Interaction. This step was the core human-robot interaction, which was quite similar in both conditions aside from the presence/absence of guidance. The interaction began when the robot’s sensors detected the participant approaching (within about 1 m for at least 2 seconds, at a roughly 90-degree field of view). The robot responded by displaying a “curious” facial expression on its screen and greeting the participant, then it activated its main menu. In both settings, the participant then navigated the on-screen menu to select a communication language and a destination (the robot offered multiple preset destinations). In the controlled trials, the researcher might verbally remind the participant of what to do next if they hesitated, whereas in uncontrolled trials the participant relied on the robot’s prompts. After the destination was selected, the robot verbally instructed the participant to follow and proceeded to lead the way. The robot provided step-by-step information or cues during navigation (e.g., “Following route to [destination]”). In the controlled scenario, per the script, the robot at some point instructed the participant to initiate a video call (simulated call) and to browse a webpage on the robot’s tablet interface (similarly to what is depicted in Figure 4). In uncontrolled scenarios, these additional tasks were optional; the robot could perform them if the participant chose, but it mainly focused on guiding. If at any time in the controlled condition the participant was unsure or took no action for ∼10 s, the researcher would step in to assist or the robot would politely terminate the interaction (“say goodbye”) and return to standby. In practice, participants generally completed the tasks without needing intervention. In uncontrolled trials, if the participant stopped interacting, the robot itself was programmed to politely end the interaction and return to its base after a short period of inactivity. Overall, this interaction step was designed to simulate real-world use of a GR: the controlled version gave an ideal, well-supported experience, whereas the uncontrolled version introduced more realistic challenges such as the user figuring out the interface alone.
- Step 3: Feedback collection. After each participant finished their interaction with the robot, we collected data via a paper-based or online questionnaire (depending on what was convenient at the venue). The questionnaire included the GRUSES items and demographic questions. A researcher was present to distribute and later collect the questionnaire (or to provide a tablet with the survey if using an online form), but participants filled it out on their own. This step was crucial for quantifying the participant’s self-efficacy and experience immediately following the interaction. Once completed, survey responses were digitized (entered into a spreadsheet) for analysis. We also recorded basic observational notes about each participant’s interaction (e.g., notable difficulties or behaviours) to contextualize the quantitative data.

#### 3.4.5 Sample

Across all trials, a total of 226 individuals participated in the survey study. The sample included university students and staff, healthcare professionals, and attendees at public tech events–reflecting a diverse mix of potential GR users. Of these participants, 110 identified as men and 106 as women (10 did not specify gender). We categorized participants into three age groups: up to 30 years old (N = 78), 31–50 years old (N = 108), and 51 and older (N = 30). Participants also reported their prior experience with using robots: the majority had no prior experience (N = 103), many had some experience (used a robot a few times; N = 114), and a small subset were frequent users of robots (N = 9). In terms of exposure conditions, 144 participants interacted with the GR in a simulated (controlled) setting, and 82 participants did so in a real-life (uncontrolled) setting. The controlled trials took place between December 2023 and February 2024, whereas the uncontrolled trials took place in May-June 2024.

All empirical work was conducted in Estonia, a relatively technology-oriented national context, and the participating organizations were drawn mainly from education, healthcare, smart-city office environments, technology events, and robotics retail settings. The survey also included relatively few participants aged 51 and older (N = 30), even though older adults and care-sector users are highly relevant for future guide-robot applications. In addition, all deployments used the TEMI v3 platform with custom software. TEMI’s tablet-based face, screen-mediated interaction, height, navigation behaviour, and proxemic defaults may have shaped user expectations and responses in ways that would differ from humanoid, voice-first, kiosk-like, or physically assistive guide robots.

#### 3.4.6 Ethics and privacy

All participants received information about the purpose of the study, voluntary participation, confidentiality, and the anonymized use of research data before data collection. Because the study posed minimal risk, involved adult participants, and did not collect personally identifiable data for analysis, the ethics committee waived the requirement for written informed consent for participation. Verbal informed consent was obtained from participants before interviews and survey participation. Two interview participants who provided written responses also provided written consent for those written responses. Interview participants additionally consented to audio recording and to the use of anonymized quotations. All data were anonymized before analysis, and no identifiable images or personal data are presented in the manuscript.

## 4 Results

### 4.1 Interview findings

In this section, we present the findings from the qualitative interview study, which form the core of our research contributions. The results are organized around the research questions on GR feature expectations, barriers to GR integration, and potential application areas (RQ3, RQ4, RQ5).

RQ3 (Feature Expectations and Development Directions): Participants across the different stakeholder groups identified numerous areas where guide robots need improvement to fit smoothly into organizational workflows. A frequently mentioned theme was the need for enhanced language support and more open developer tools. As one library employee noted, *“Better language support, improved SDK to control the robot functions, more standards”* [P6]. In our study context (Estonia), having the robot fluently speak the local language was seen as critical; distributors confirmed that *“quite often a customer’s wish is that I want a robot attendant that speaks Estonian”* [P2].

**FIGURE 5:**
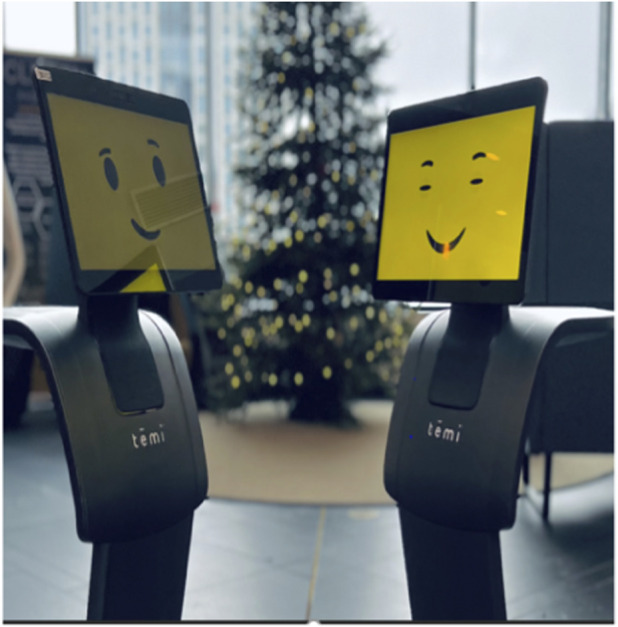
TEMI displaying facial expressions on its screen, used to convey emotions during interaction.

Another major expectation was more human-like expressiveness in the robot’s behaviour. Participants called for advancements in the robot’s facial expressions and gestures to create more engaging interactions. One interviewee suggested, *“Develop robots with a friendlier and more human-like appearance and behaviour, making them more pleasant and less intimidating to* users” [P8]. Others emphasized specific cues: *“A smiley face or a questioning look on the screen helps build trust”* [P20]. Indeed, many users appreciated when the robot attempted eye contact or showed emotion: *“I liked it when the robot moves the screen, so it looks like it is looking at you. It made the interaction feel personal”* [P21]. Figure 5 illustrates the kind of expressive “emotions” displayed by the TEMI robot, which participants found endearing but believed could be expanded further.

Appropriate proxemics (the robot’s use of personal space) also emerged as important. Users reported discomfort if the robot came too close (within about half a meter) without warning, often causing them to step back instinctively. Conversely, if the robot kept too large a distance, users were sometimes unsure if it was engaging with them. A hospital employee praised the robot’s default behaviour: *“It kept a polite distance, not too close, not too far, which made people feel safe interacting”* [P16]. However, there were instances where abrupt movements undermined this comfort. A librarian recounted, *“It kind of did a quick turn without warning–I actually jumped back because I did not expect that”* [P19]. Users suggested that robots clearly indicate their intentions before moving–for example, by giving a verbal cue like “Follow me now” before starting to roll forward. Such a prompt would prepare users and synchronize the robot’s verbal and nonverbal signals, preventing surprise or confusion.

Participants also debated the movement speed of the robot. Generally, TEMI’s pace was set to a moderate walking speed, which most found acceptable, but context mattered. Some users in busy workplaces wanted a faster response: *“When I’m in a hurry, having to follow the robot feels very time-consuming. It could move at the same pace as m*e” [P9], said one corporate office worker. On the other hand, in an elderly care context, a staff member noted, *“The robot needs to move slowly because older people’s attention spans are short, and they might not notice the robot moving right away”* [P24]. In fact, if the robot suddenly accelerated, it could startle users: *“The robot suddenly started moving quickly. I did not expect it and thought I had somehow triggered it by mistake”* [P16]. A common suggestion was for the robot to adapt its speed to the user or environment–slowing down in crowded or elder-care environments and having an option to move faster when guiding someone who is clearly in a hurry.

Beyond behaviour and interaction improvements, customization and modularity were highlighted as priorities for future development. Many participants expressed the desire for robots that could be easily tailored to specific organizational needs or personal preferences. *“Users should be able to easily customize the robot’s appearance and behaviour. Modular design allows for easy addition or replacement of components,”* explained one tech-savvy participant [P8]. For example, a library administrator imagined adding a module to scan RFID tags on books, whereas a hospital manager thought about integrating a temperature sensor or ID card reader into the robot. Distributors echoed this need: clients often ask if the robot can be modified for the particular tasks. The consensus was that a one-size-fits-all robot is less useful than one that can be configured with the right tools (software or hardware add-ons) for the task at hand.

RQ4 (Barriers to Effective Use and Behavioural Expectations): Participants identified a range of barriers that currently prevent users from being fully effective with GRs. These barriers fell into several categories–aligning with the themes from our analysis.

First, there were technological barriers. Users and managers reported that today’s GRs still have “rough edges” in terms of reliability and capabilities. One distributor frankly stated, *“These products are so raw; they are not re*ady” [P2], referring to how immature some commercial robots still are. Frequent malfunctions or errors were noted: lost network connections, freezes, or navigation failures erode user confidence quickly. Limited navigation ability in complex or cluttered environments was a common complaint (e.g., the robot sometimes got stuck or took inefficient routes). Insufficient verbal and nonverbal language support was also seen as a barrier–outside of major languages, the robot’s speech and recognition capabilities were lacking, and even in English or Estonian, its tone or inflection sometimes seemed unnatural or hard to hear in noisy environments. A distributor summarized a key issue: *“If the user interfaces and functions of robots are not intuitive, it can lead to frustration for users and reduce motivation to use the robot”* [P8]. Indeed, some end-users criticized the tablet interface design as not very user-friendly for those who are not tech-savvy, creating an accessibility gap.

Turning to user-related barriers, psychological factors like technophobia and low confidence emerged prominently. Some participants observed colleagues or visitors who hesitated to approach or touch the robot: *“Some people have a fear of technology. They are afraid of touching things they do not know*” [P12], a librarian noted. This fear of “breaking something” or making a mistake can prevent people from even attempting to use the robot. Fear of doing something wrong with the robot was mentioned, especially in uncontrolled settings–users worry they might get stuck or not know how to recover if they press the wrong button. Privacy concerns also surfaced repeatedly as a barrier to engagement. People were unsure what the robot’s sensors were recording. Some visitors would avoid the robot out of an unease of being watched: *“No, I do not want to interact with it because I do not know who’s watching from inside or who’s monitoring”* [P9] was a sentiment expressed about camera concerns. Similarly, others questioned, *“How can I be sure I’m not being photographed or filmed by a robot?”* [P18]. Such privacy anxieties can severely limit willingness to use GRs, especially in cultures or contexts where surveillance is sensitive.

Context-dependent proxemics issues were mentioned as well. For tasks that required a person to press the robot’s screen or follow closely, participants expected the robot to adjust its distance appropriately. One person gave an example: *“The robot could come closer to allow pressing the screen but kept a greater distance with customers just passing by*” [P24]. In crowded areas, users wanted the robot to be smart about navigating around people and maintaining personal space. *“The robot should be able to quickly assess how to pass a person in a busy space,”* suggested another interviewee [P24]. Additionally, a few participants raised cultural norms as an important consideration: if the robot operates in multicultural settings, it should be aware that personal space and social cues can vary. *“It must be taken into account … it causes discomfort when you have to change your habits, and it limits the use [of the robot],”* one participant [P23] said, implying that if a robot violates local social norms (e.g., how close to stand, whether to bow or not, etc.), people might reject it.

Interestingly, our interviews revealed that many users initially had limited expectations for a robot’s social behaviour–they did not expect a machine to follow human conventions. However, once they interacted with the GR, they realized they did appreciate human-like non-verbal behaviour. For example, a university employee mentioned that seeing the robot’s “gaze” or at least some indication of attention was very important: *“You are used to talking to a person face to face; even when you talk to a machine, you are still not sure in the back of your head whether it heard you [if it does not show signs of listening]”* [P20]. This suggests that users need feedback from the robot (like looking at the speaker or a text on screen such as *“I’m listening … ”*) to feel confident that the interaction is working. Because of these subtle uncertainties, several respondents proposed that users be given short training or orientation on how the robot behaves. They felt that, in addition to a technical manual, there should be a simple guide to the robot’s behavioural patterns–essentially “What to expect when interacting with me.” As one participant put it, *“The robot could say: I’ll tell you about myself–how I behave, move, why I do something, etc. What should you consider when communicating with me? What I can do well, and where I still need practice”* [P23]. Another said, *“I would even expect prior training or instructions”* [P20] before using a robot as part of your job. This highlights that building user self-efficacy might require preparatory orientation, not just good design of the robot alone.

A specific question we posed was how participants felt about the possibility of a robot physically touching them (or vice versa). The consensus was clear: a robot should not initiate touch with a person under normal circumstances. *“I do not dare shake the robot’s hand, because I know that there is no human brain but a machine,”* one user remarked [P20], reflecting a discomfort with anthropomorphic gestures like handshakes from a robot. However, participants were more open to scenarios where the human initiates or consents to touch. For instance, a robot could offer a handle or arm for a person to hold onto if they need balance support. *“It is not that it holds on to you, but the robot could have a place to lean on, if you wish. It offers the possibility to lean on, rather than holding on [to you],”* explained [P26]. In a caregiving context, an interviewee reacted positively to the idea of a robot offering support: *“…if a person has difficulty walking, a robot could offer them help so that the person can lean on the robot’s arm”* [P24]. Thus, any physical interaction must be on the user’s terms–the robot can offer but not impose contact. This insight is important for future GR designs (e.g., whether to include physical guiding or haptic feedback).

Participants from the distributor and manager groups pointed out a crucial organizational barrier: lack of support from top management and unclear role definition for the robot within the organization. One distributor noted that clients often do not have a plan for the robot after purchase: *“A common question is how to use the robot effectively in specific situation*s” [P8]. Similarly, a manager admitted, *“We could not find enough activities for the robot or explain it to our employees”* [P9]. Another manager echoed, *“We need to better explain the role of the robot in our department to our employees”* [P19]. Without strong leadership support and a clear integration plan, staff may ignore the robot or use it inconsistently, undermining its value. This points to the need for change management when introducing GRs–staff should be briefed on why the robot is there, what it should be used for, and how it benefits their work, to avoid confusion or resistance.

**FIGURE 6:**
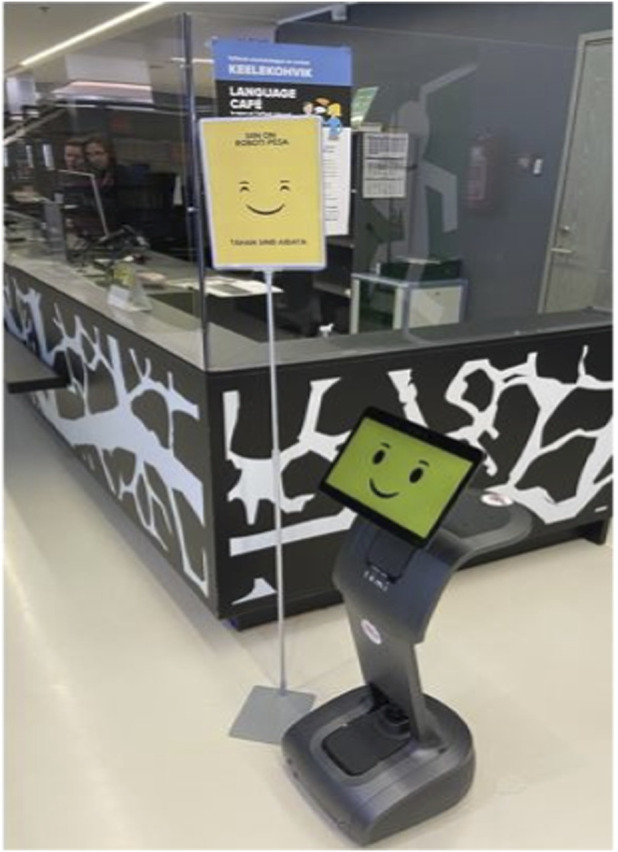
TEMI V3 robot working in TalTech library, guiding a user to a section–an example of GR deployment in an organizational setting.

RQ5 (Application Areas and Organizational Adjustments, similar to what is depicted in Figure 6): Our findings suggest that guide robots are well-suited for certain kinds of tasks in organizations, but not all. Participants generally agreed that GRs excel at routine, repetitive tasks that do not demand emotional intelligence or complex decision-making. These include guiding visitors through a facility, providing standard information, assisting with simple logistics, such as delivering small items or documents, and managing inventory queries. For instance, a distributor mentioned that some GRs are already used to *“serving food/snacks in restaurants or events”*, implying tasks that involve moving items around predictably [P8]. A librarian participant hoped, *“If TEMI could guide the reader to the exact spot–say, ‘Look here, there’s your book’ – that would be great”* [P12]. In a hospital scenario, one idea was: *“While the human is waiting, the robot might say: ‘Take out these documents that need to be submitted’”* [P3], meaning the robot could instruct patients on preparatory steps in a process. These examples illustrate how robots might take on assistant roles to free up staff time and streamline services.

Conversely, participants noted that GRs are unsuitable for tasks requiring empathy, complex judgment, or high adaptability. Anything that demands human understanding, emotional support, or intricate problem-solving is not (yet) in the robot’s wheelhouse. This underscores the importance of clearly defining the roles of GRs within organizations: they should augment humans by handling straightforward tasks, while humans continue to handle the nuanced ones. One participant emphasized that making this role clarity explicit to everyone is key to successful adoption.

From an organizational perspective, the introduction of GRs raised concerns among staff about workloads and job security. Several staff members initially feared the robot might increase their work or even replace them. One participant shared an incident: *“The receptionists did not want to show their faces during calls. The employee should understand that TEMI helps them* [r*ather than replaces them]”* [P9]. This indicates that some staff avoided using a new video-call feature through the robot, possibly out of discomfort or fear of being monitored. Addressing these fears requires communication that the robot is a tool to assist, not a threat to jobs, and possibly redesigning workflows so that employees clearly see a reduction in their burden.

Privacy and security improvements were also deemed essential to build trust in GR deployment. As mentioned in RQ4, users worry about data and surveillance. Both end-users and managers recommended transparency in what data the robot collects and why. As one participant succinctly put it, *“Transparency about data collection is necessary to alleviate fears”* [P9]. This might involve giving users some control (like a privacy mode) or providing clear signage or verbal assurance about any cameras or recordings.

Participants had various opinions on technical settings like the robot’s speed (as discussed), but generally they agreed that the appropriate behaviour might vary by use case. For example, in a hospital, the urgency might dictate quicker movement, whereas in a museum a slower, leisurely pace is preferable. One interviewee described internal debates: *“There are different opinions in the hospital. Some thought it moved too slowly when needing to reach another ward quickly; but if it is just patrolling, then it can move at a leisurely pace”* [P26]. This again highlights the need for context-aware adaptability in GRs, either through configuration or intelligent sensing.

As a new suggestion beyond our original questions, participants imagined future enhancements where GRs could take on more proactive roles. One intriguing idea was equipping the robot with the ability to detect human behaviour anomalies and offer help unprompted. For instance, in large buildings like airports or hospitals, a GR could notice if someone looks lost (e.g., standing in one spot and looking around for a few minutes) and then approach to ask if assistance is needed. One interviewee described it: *“A person does not understand where to go. They might stare at the directory board for 3 min. The robot could notice that and go ask if it can help”* [P20]. This kind of initiative could greatly increase the robot’s value as a guide. Participants noted that robots could also perform helpful tasks like reminding patients about medications or schedules (a distributor said robots are effective at *“providing reminders to patients to take their medications or guiding people in a large hospital”* [P8]). These forward-looking ideas suggest that once basic issues are solved, users are excited for GRs to have more autonomy in assisting people.

In summary, the qualitative study’s findings emphasize that while GRs are currently best at routine guidance and information tasks, their acceptance in organizations hinges on improving technical reliability and behaviour (especially nonverbal interactions), addressing user fears (through better design and training), and making organizational adjustments (clear roles, infrastructure, support). Users and stakeholders have a clear vision of both what the robots should ideally do and what needs to be in place around the robots for them to succeed.

### 4.2 Findings from the survey study

We now turn to the results of the preliminary self-efficacy survey, which assessed GR users’ confidence levels and how these were affected by personal factors and usage context. As noted earlier, these findings should be interpreted as exploratory since the GRUSES scale is newly developed and this stage was intended primarily to inform and complement the qualitative insights.

Before addressing RQ1 and RQ2 directly, we provide an overview of the scale’s properties. Supplementary Table 2 (see Supplementary Appendix 2) lists the GRUSES item means, which ranged from approximately 4.7–5.8, the overall mean was M = 5.40. Participants responded on a 7-point scale (1 = strongly disagree, 7 = strongly agree). The overall mean indicates that, on average, participants tended toward agreeing with positive statements about their ability to use the GR. In fact, we observed a generally positive response bias–item means ranged from about 4.7 to 5.8 on the 7-point scale, suggesting most users felt reasonably confident and comfortable using the robot. This could reflect curiosity and enthusiasm in the controlled trials, but it also raises the possibility that some participants gave optimistic ratings. The high mean scores indicate a possible positive response bias and ceiling effect. In the available pilot output, several core items had high means and negative skewness, for example, complexity/ease of communication (M = 5.89, skewness = −1.0), clarity (M = 5.91, skewness = −0.8), security (M = 5.69, skewness = −0.7), suitability (M = 5.76, skewness = −0.7), and comfort (M = 5.50, skewness = −1.0). These values suggest that the scale may be less sensitive to variation among users who already feel moderately or highly confident.

We assessed the internal reliability of the GRUSES scale with Cronbach’s alpha that was approximately 0.9. The exploratory factor analysis supported using the scale as a single composite score in this exploratory study. The scale showed high internal consistency, meaning the items were strongly interrelated and likely measuring a single underlying construct (user self-efficacy in using a GR). The exact reliability analysis is summarized in Supplementary Table 2 (Supplementary Appendix 3). An alpha of 0.90 exceeds the common threshold (0.8) for research instruments, suggesting that GRUSES provides reliable scores for our sample. We also took steps to establish content validity (by involving HRI and psychometrics experts during development) and face validity (through pilot feedback, it appeared to measure what it intended to).

To examine the scale’s structure (construct validity), we performed an exploratory factor analysis. We initially hypothesized two subscales (operational vs. affective efficacy), but the factor analysis results indicated that the scale was essentially unidimensional–all items loaded on one dominant factor. In other words, participants who felt confident in one aspect (like operational use) also tended to feel confident in the other aspects (like comfort and safety), so it made sense to treat the GRUSES score as one overall self-efficacy measure. (See Supplementary Table 2 in Supplementary Appendix 4 for factor analysis details.). Consequently, for this study we used the scale as a single composite measure of GR self-efficacy.

At this stage, the article reports content validity based on expert item generation, face validity based on pilot feedback, internal consistency based on Cronbach’s alpha, and preliminary factor-structure evidence. However, convergent and discriminant validity were not tested because no established comparator instrument, such as RUSH-3 or SE-HRI, was administered to the same participants. Accordingly, the quantitative results are treated as preliminary and hypothesis-generating rather than as evidence from a fully validated scale.

Based on the survey results, we can answer RQ1 and RQ2 as follows:

- RQ1 (Self-efficacy differences by gender, age, experience): Overall, participants rated their ability to use the guide robot quite positively (mean around 5.4 on the 7-point scale), indicating a generally high self-efficacy. We found that previous experience with robots was a significant differentiator in self-efficacy scores. As expected, participants with prior experience using robots reported higher self-efficacy (average M ≈ 5.69) than those without prior experience (M ≈ 5.09). This difference was statistically significant (independent samples t-test, t (219) = 3.47, p < 0.01). In contrast, we did not find significant differences in self-efficacy between different age groups or between men and women in our sample–any small differences in means were not statistically meaningful. It appears that regardless of age or gender, people’s confidence in using the GR was more uniformly high, but prior hands-on experience did give some participants an edge in confidence. These results underline that familiarity with technology (especially robots) can boost users’ self-efficacy, whereas demographic factors like age or gender alone did not show an effect in this context.
- RQ2 (Effect of controlled vs. uncontrolled context on self-efficacy): We observed a clear context effect. Participants who used the robot in the controlled, guided environment reported significantly higher self-efficacy (mean M ≈ 5.68) compared to those who used it in an uncontrolled, real-life scenario (mean M ≈ 5.05). This difference was statistically significant (t (219) = 4.91, p < 0.01). In practical terms, interacting with the GR under ideal, supportive conditions led to more confidence, while the challenges of the real-world setting made some participants feel less efficacious. This pattern held true not just for the overall score but was also reflected on individual items and any attempted subscales: the situational context had a broad impact on how users felt about their capabilities. The drop in self-efficacy from controlled to uncontrolled scenarios suggests that even if users initially feel confident (perhaps due to novelty or supportive introduction), that confidence can falter when they face the complexities of a real environment without guidance. We interpret this as evidence that contextual factors (like unpredictable interference, absence of immediate help, greater stakes of failure) play a significant role in user confidence. Differences in GRUSES scores may reflect not only the level of researcher support and interaction autonomy, but also contextual differences between venues, user groups, and recruitment situations. For this reason, the results are described as an observed association between interaction context and self-efficacy, not as causal evidence that the setting alone produced the difference.

In summary, the survey’s results reveal that prior exposure to robots increases user self-efficacy, and that users’ confidence can be considerably higher in test/demo conditions than in authentic usage conditions. These findings provided important context for our interviews: they signalled, for example, that organizations should pay attention to training novice users (since experience matters) and that positive attitudes seen in trials may not directly translate to unassisted usage. The survey findings were used to shape the interview questions–for instance, we probed interviewees about why they thought people might feel less confident in real settings and what could be done to bridge that gap. However, it is important to note that the survey results are preliminary. The GRUSES scale is still in an early validation phase, and the positive bias observed suggests that future work (discussed below) should refine the instrument to capture a wider range of user feelings, including potential negative experiences, to avoid ceiling effects.

### 4.3 Integration of survey and interview findings

The survey and interview findings are integrated here not only by alignment, but also by extension and reinterpretation. At the user level, the survey indicated that prior robot experience was associated with higher self-efficacy. The interviews extend this finding by showing that “experience” is not only repeated exposure to a robot, but also a form of interaction literacy: users need to know what the robot can do, how it signals attention, how close it will come, how it moves, and what to do when interaction does not proceed as expected. Thus, the qualitative findings reinterpret the quantitative experience effect as a training and sense-making issue rather than merely as a demographic or familiarity effect.

At the robot and interaction level, the survey showed generally positive GRUSES scores and possible ceiling effects. The interviews qualify this result by revealing that positive self-efficacy ratings can coexist with fragile confidence. Participants often evaluated the robot favourably but still described concrete sources of uncertainty, including navigation reliability, abrupt movement, unclear gaze direction, language limitations, privacy concerns, and uncertainty about error recovery. The qualitative data therefore suggest that the survey captured general confidence after interaction, whereas the interviews exposed situational conditions under which that confidence may weaken.

At the organizational level, the survey showed lower self-efficacy in uncontrolled real-life settings than in controlled guided settings. The interviews reinterpret this difference as more than a simple contrast between two technical environments. Users’ confidence declined when immediate support was absent, when the robot’s role in the workflow was unclear, when staff were unsure how to intervene, or when users felt publicly observed while using the robot. Together these two strands show that guide-robot acceptance depends on the joint configuration of user readiness, robot capability, and organizational readiness. The survey identified where confidence differed, while the qualitative findings explain why the differences may occur and how organizations can reduce them through training, role definition, legible robot behaviour, privacy transparency, and support routines.

## 5 Discussion

The worsening shortage of skilled workers in key service sectors (retail, healthcare, education, etc.), especially in aging societies, has fuelled interest in adopting robotic technologies as a partial solution. Over the past decades, technologists have been optimistic about integrating robots into service organizations, yet in practice, such integration has rarely moved beyond experimental or pilot stages. Our study underscores that successful adoption of robots in socially structured environments entails much more than procuring advanced hardware and deploying it on site. As our theoretical review highlighted, adopting robots is a complex social process that must draw on interdisciplinary research–combining insights from social sciences (user attitudes, organizational behaviour, etc.) with technological development (robot capabilities, AI behaviours) to ensure robots can truly function in human-centric settings.

In this research, we focused on GRs as an example of SSRs and examined both technological features and human factors influencing their acceptance in organizations. A central human factor we investigated was user self-efficacy, drawn from Bandura’s theory, which we argue is critical in HRI within workplaces. Our preliminary findings indeed show that certain demographic and experiential factors shape user self-efficacy. Specifically, prior exposure to technology–in this case, having used robots before–was associated with higher self-efficacy among GR users. This aligns with prior research on technology adoption which suggests that more experienced or tech-savvy users are quicker to embrace new systems (Nomura and Kanda, 2021). It indicates that when introducing GRs, organizations should consider the characteristics of their user groups (age, tech experience). For example, younger staff or those who have seen or used robots before might become early adopters or “robot champions” in the workplace, whereas extra support might be needed for those with no prior exposure. Interestingly, our data did not show a significant gender difference in self-efficacy, which is a positive sign–at least in this sample, women and men were equally confident in using the technology, contrary to some stereotypes regarding technology use. This finding agrees with some recent studies that find gender gaps in technology perception are narrowing (Moseley and Mead, 2023; Draxler et al., 2023) though it contrasts with others that still find differences (Jones et al., 2025). It may be context-dependent and merits further study.

Our survey also revealed a situational effect: self-efficacy drops in real-life usage conditions compared to controlled trial conditions. In controlled settings (which often resemble demos or training sessions), participants felt quite confident, but in the uncontrolled, “live” environment, their self-efficacy was significantly lower. This result serves as a cautionary tale against over-interpreting positive user feedback from lab tests or short pilot programs. It suggests that many optimistic conclusions from previous laboratory-based HRI studies–which often report high user interest and acceptance–may not fully hold up in everyday practice. Our finding echoes observations by Hung et al. (2019), who noted that users often exhibit higher confidence and comfort in controlled experiments due to the predictable environment and the availability of immediate support. When those safety nets are removed, users may encounter unexpected issues or simply feel a greater sense of responsibility (and thus anxiety) in making the robot work properly. In our case, realizing this discrepancy was one of the motivations for conducting a qualitative study. We saw that *“trying GRs just for fun”* versus *“using them as part of routine workflows”* led to different user experiences, so we set out to understand *why* and *how to mitigate* that gap.

The decline in self-efficacy from controlled to real-world use can be interpreted through several psychological mechanisms. In the controlled condition, the presence of a researcher reduces uncertainty, provides immediate corrective feedback, and creates a low-stakes mastery experience. In the uncontrolled condition, users must interpret the robot’s prompts, recover from possible errors, and complete the task under public visibility without immediate assistance. This may increase cognitive load, reduce perceived control, and heighten anxiety about making a mistake. The available qualitative evidence from related TEMI deployments also suggests that unexpected queries, technical glitches, bystander observation, and lack of immediate support can make users attribute interaction problems to their own inability rather than to the robot or context (Leoste et al., 2024). Thus, the controlled-to-real-world decline should be understood not only as a usability issue but also as a self-efficacy mechanism shaped by support, predictability, and error-recovery opportunities.

The importance of gaze, proxemics, facial expression, and predictable movement can be interpreted through social presence and human-likeness in interaction design. When the robot turns its screen toward the user, keeps an appropriate distance, signals its intention before moving, and displays readable facial expressions, users can more easily treat it as an attentive interaction partner. These cues do not need to make the robot fully human-like; rather, they make the robot’s state and intention legible. This legibility supports self-efficacy because users can predict what the robot is doing and how they should respond. Conversely, abrupt movement, unclear gaze direction, or poorly timed feedback can reduce perceived control and create uncertainty. The findings therefore suggest that nonverbal behaviour contributes to guide-robot acceptance through perceived social presence, predictability, and interactional clarity (Saunderson and Nejat, 2019; Leoste et al., 2024).

The findings from the interviews provide richer insight into the barriers and enablers of GR acceptance. One of the significant contributions of this study is highlighting how improving certain robot behavioural features can positively influence user self-efficacy and trust, thereby easing integration. Participants pinpointed gestures, facial expressions, emotional tone, speech patterns, movement styles, and other nonverbal cues as important factors that affect how comfortable they felt using the robot. Our results show that when GRs behave in ways that align with human social expectations (e.g., maintaining a polite distance, making “eye contact,” moving predictably), users gain confidence and are more willing to engage. This finding extends prior work that has called for human-aware robot behaviours by demonstrating the direct link to self-efficacy: not only do such behaviours make interactions more pleasant, but they also actually empower users by making them feel more in control and capable. Conversely, when the robot’s behaviour violates social norms or behaves erratically, users’ confidence drops and they may even abandon the interaction (as seen in some uncontrolled trial anecdotes). Thus, from a design perspective, socially intelligent behaviour in robots is not just about user satisfaction but also about user efficacy.

Our respondents also emphasized the importance of user training and support mechanisms as complementary to technical improvements. Many interviewees argued that giving users a bit of orientation–whether via formal training sessions or built-in robot tutorials–can build the necessary self-efficacy for sustained robot use. This aligns with Bandura’s idea that mastery experiences (successful practice) build efficacy, and vicarious experience or verbal persuasion (being guided or reassured) can also help. In practice, organizations should treat the introduction of GRs similar to introducing a new software system: provide documentation, quick-start guides, and responsive support, especially in the early phase. We found that users appreciated knowing help was available (in the controlled trials) and that boosted their confidence; by extension, if organizations ensure ongoing technical support or a “robot helpdesk,” users might be more inclined to persevere through initial difficulties.

These insights underscore a broader point: there is a complex interplay between human and technological factors in determining HRI outcomes. It is not enough to fix either the human side (train the users) or the technology side (improve the robot) in isolation–both must advance together for successful implementation. Our study suggests such interplay: we saw that demographic/human factors (experience, attitudes) influence how people approach the robot; the robot’s design (behavioural cues, interface) influences how people feel and react; and organizational context (management support, clarity of the robot’s role) influences whether the conditions for fruitful interaction are present. All these factors loop back to affect user self-efficacy and acceptance.

Comparing our findings to existing literature: earlier HRI studies have extensively discussed trust, perceived usefulness, and ease of use (e.g., in terms of the Technology Acceptance Model), but self-efficacy has been less of a focus in robotic contexts. Our work contributes by explicitly focusing on self-efficacy and showing how it is shaped by and also shapes these other factors. For instance, trust in technology (a commonly studied construct) is likely bidirectional with self-efficacy–as users feel more capable, they trust themselves and the robot more, and as the robot proves trustworthy, it reinforces their self-confidence. We also resonate with studies such as (Savela et al., 2022) which noted that familiarity breeds acceptance–our data on prior experience confirms that. We add nuance by showing that even without prior experience, certain design choices can make first-time users feel capable (some of our controlled environment users had no experience but still rated high efficacy, presumably due to the supportive design of the interaction). In organizational HRI, self-efficacy therefore serves as a distinct analytical bridge between robot-level affordances, user-level confidence, and organization-level readiness, explaining not whether a robot is judged useful or trustworthy, but whether users feel capable of engaging with it competently within real work routines.

It is worth noting that organizational factors emerged in our study as fundamental but are often underrepresented in HRI research. Many studies treat the environment as a static background, whereas our interviews with managers and distributors revealed issues like lack of clear strategy for robot usage, staff resistance due to misunderstandings, and insufficient maintenance support. These are not robot “problems” *per se*, but they can make or break a deployment. Our results thus align with frameworks that call for socio-technical integration efforts, for example, ensuring that the introduction of a robot is supported by organizational change management. Participants repeatedly mentioned that if management does not actively endorse the robot and define its role, employees either ignore it or use it inconsistently, which in turn means the robot does not deliver value. This highlights a practical recommendation: organizations need to treat a GR like introducing a new team member–with announcements, role definitions, and processes to incorporate it–rather than like a gadget that is just put in the corner to see what happens.

The organizational barriers identified in the interviews can be interpreted through socio-technical systems theory. A guide robot is not adopted only as a technical artefact; it becomes part of an interdependent system of people, tasks, infrastructure, routines, and managerial expectations. Technical reliability and navigation are necessary, but they do not by themselves create organizational value. Value emerges when the robot’s role is defined, staff know when and how to involve it, users understand what the robot can and cannot do, and the physical and digital environment supports its operation. From a change-management perspective, guide-robot deployment therefore requires communication, role definition, staff preparation, and feedback loops rather than one-time installation. This interpretation explains why unclear management support and weak role definition appeared as central barriers in the qualitative findings (Zeller and Dwyer, 2022; Bostrom and Heinen, 1977; Kotter, 1996).

### 5.1 Practical implications

Based on the integrated findings, the following recommendations should be prioritized. First, organizations should define the robot’s role before deployment, including which tasks the robot performs, when staff should intervene, and how users are informed about the robot’s purpose. Without role clarity, even technically capable robots may remain underused. Second, basic technical reliability, navigation accuracy, language support, and privacy transparency must be ensured because failures in these areas directly undermine user confidence. Third, users and staff need short orientation and accessible support materials, especially for first-time users and public-facing employees who may need to assist others. Fourth, robot behaviour should be made legible through appropriate gaze, proxemics, speed, pre-movement signals, and expressive feedback. Fifth, organizations should adapt the physical and digital environment, including routes, signage, charging, and integration with relevant information systems. This ordering should not be read as an empirical ranking of effect sizes, because the study was exploratory and did not statistically differentiate the relative importance of these recommendations. It reflects implementation logic derived from the integrated findings: role clarity and reliability are prerequisites for meaningful use, training and legible robot behaviour help users build confidence during interaction, and infrastructure adaptation and feedback loops support sustained organizational use over time.

### 5.2 Limitations

Given the scope and design of our study, there are several limitations to acknowledge. First, the sample size in the qualitative stage (N = 26), while providing depth, is still relatively small and drawn from specific sectors (education, healthcare, etc.) in one country. This means the themes we identified, though salient, may not cover all possible issues or may not generalize to other cultural or organizational contexts. There is a potential self-selection bias as well: the individuals who agreed to participate might be those more interested in or positive about robotics, which could skew the findings towards highlighting improvement possibilities rather than outright rejection. We attempted to include some sceptical voices (indeed, some participants voiced strong concerns), but a broader outreach might have found individuals unwilling to even participate because of disinterest or negativity toward robots.

Second, while our mixed-method approach is a strength, the two stages were unbalanced in weight–the survey was preliminary and the scale still undergoing validation. The GRUSES scale we developed is in early stages and was tailored to our scenario; thus, the quantitative results should be interpreted with caution. The positive bias in responses suggests that perhaps some items were phrased in a way that elicited agreeability, or participants in controlled trials felt compelled to give high ratings (social desirability). Future research should refine this instrument: we plan to assess its convergent validity (does it correlate as expected with related constructs like general self-confidence or tech anxiety?) and discriminant validity (does it avoid overlapping too much with, say, general attitude toward robots?). We also realized that our scale might need items specifically addressing obstacles or frustrations, to capture negative experiences; currently it might be too focused on positive aspects, which could explain the skew. Our findings should be interpreted as indicating generally positive self-efficacy tendencies rather than finely differentiated levels of confidence. Future GRUSES versions should include items that capture hesitation, uncertainty, error recovery, frustration, and discomfort, and may also include carefully designed negatively worded or difficulty-sensitive items to reduce acquiescence bias.

Third, our experimental design for RQ2 – comparing controlled vs. uncontrolled conditions–was not a pure randomized experiment but rather quasi-experimental, since participants were not randomly assigned. There could have been differences between the groups aside from the condition (for example, those at the tech fair might have been more tech-savvy than those at the library). We mitigated this by mixing venues for both conditions as much as possible and by having a fairly large N, but uncontrolled factors could have influenced the outcomes. For instance, the uncontrolled trials happened later in the timeline; by then, maybe the novelty had worn off a bit or we (researchers) were less actively enthusiastic in inviting participants, etc. Longitudinal effects were not measured; ideally, one would want to measure the same individuals’ self-efficacy first in a demo and later after actual usage, to really see the drop within-subject. Because of various limitations, the results are offered as evidence of an observed association between the interaction context and self-efficacy, and not that of causal evidence that the setting alone produced the difference.

Finally, the cultural context (Estonia) is relevant–Estonia is quite tech-forward (e.g., e-government, delivery robots on sidewalks are not unheard of), so baseline receptiveness might be higher than in places where robots are rarely seen. This could have influenced the generally positive tone of responses. Conversely, some findings like not fearing job loss too much might not hold in other labour markets. We acknowledge that our findings, while internally valid, remain limited and need to be tested with older and less technology-oriented populations, in other national contexts, and across multiple robotic platforms.

### 5.3 Future research directions

This work opens several avenues for further studies. One immediate next step is to continue validating the GRUSES scale. We plan to conduct larger surveys with more diverse populations, including settings where the robot is used over longer periods. Testing the scale in multiple countries would also help ensure it is capturing the same constructs across cultures (for example, perceptions of safety and comfort might differ culturally). We are also interested in testing a short-form of the scale for practical use–for organizations to quickly gauge user self-efficacy with minimal burden.

Another future direction is to conduct longitudinal studies. Many participants only had brief exposures to the GR. It would be valuable to see how self-efficacy and acceptance evolve as people work with a robot regularly over weeks or months. Does self-efficacy naturally increase as familiarity grows (likely yes), and does that plateau or even dip if, say, a malfunction happens that breaks trust? Longitudinal data could also show how training interventions impact self-efficacy retention over time.

Additionally, future studies should differentiate more clearly between real-world and laboratory settings in their analysis and discussion of results. Our work showed one way to simulate “real” vs. “lab” usage; building on this, researchers could design experiments where the only difference is the presence of help or the framing of the task (optional vs. mandatory) to isolate which aspect causes the confidence drop.

Another interesting line of research would be exploring adaptive robot behaviour driven by AI that can sense user discomfort or uncertainty. Some participants basically suggested this–e.g., the robot noticing a confused person and offering help proactively. If a robot could detect that a user is struggling (perhaps by inactivity or facial expression analysis) and then adjust its behaviour (slowing down, offering more guidance, switching to an “assist mode”), would that improve the user’s self-efficacy in the moment and subsequent interactions? Such adaptive systems could personalize support, which ties in with Bandura’s idea of guided mastery–the robot itself could become the guide in teaching the user, not just physically guiding them in space.

From an organizational perspective, future work could also examine organisational change-management strategies for robot integration. For example, study two similar organizations where one introduced a GR with a comprehensive staff engagement program and the other just deployed the GR without much support. Compare outcomes in acceptance, usage rates, and employee attitudes. This would provide evidence on the best practices for organizational adoption of robots.

Ethical considerations also merit more research. Privacy and autonomy concerns came up in our study; exploring how different transparency measures (like a “privacy mode” indicator on the robot, or providing users control over data) affect acceptance could guide ethical design. Additionally, as robots get more capabilities (e.g., anomaly detection as suggested), ensuring they respect human dignity and agency will be crucial for acceptance. For instance, a robot approaching someone to help could be seen as great by one person but as intrusive by another; research on consent and social norms for robot-initiated interaction would be valuable.

## 6 Conclusion

In conclusion, our study highlights that integrating guide robots into organizational workflows is not just a technical deployment, but a socio-technical transition that involves user confidence, robot design, and organizational readiness. By treating user self-efficacy as a focal lens, we were able to uncover how these dimensions interact. We hope that our findings encourage both researchers and practitioners to devote as much attention to the “human side” of HRI–training, support, social behaviour–as they do to improving the robots’ technical capabilities. With a balanced approach, guide robots can indeed become effective partners in service organizations, augmenting human labour and enhancing experiences for users and customers.

## Data Availability

The raw data supporting the conclusions of this article will be made available by the authors, without undue reservation.

## Supplementary material

SUPPLEMENTARY TABLE 1 Thinking about your experience today, how do you rate your interaction/communication with the robot? Think about the tasks you solved in cooperation with the robot today. How would you assess the completion of these tasks (such as moving from one room to another, etc.) together with the robot? While you were working with the robot, other people were also present in the room. What was their attitude towards the interaction between you and the robot and acting together?.

SUPPLEMENTARY TABLE 2 Descriptive statistics of the GRUSES scale items.

SUPPLEMENTARY TABLE 3 Reliability of the GRUSES scale.

SUPPLEMENTARY TABLE 4 Factor analysis of the GRUSES scale items.

## References

[B1] Adetayo A. Abwage K. Oduola T. (2023). Robots and human librarians for delivering library services to patrons. Ref. Libr. 64, 69–84. 10.1080/02763877.2023.2183303

[B2] Almokdad E. Lee C. H. (2024). Service robots in the workplace: fostering sustainable collaboration by alleviating perceived burdensomeness. Sustainability 16 (21), 9518. 10.3390/su16219518

[B3] Ang E. Bejleri A. Tantisira B. Van de Velde A. (2024). Considerations for the future of social robots and human–robot interactions. OxJournal Comput. Sci. Available online at: https://www.oxjournal.org/the-future-of-social-robots-and-human-robot-interactions/. (Accessed March 29, 2026)

[B4] Arreghini S. Abbate G. Giusti A. Paolillo A. (2024). “A service robot in the wild: analysis of users intentions, robot behaviors, and their impact on the interaction,” in Proceedings of the IEEE/RSJ International Conference on Intelligent Robots and Systems (IROS).

[B5] Aryati A. Armanu M. (2023). The influence of self-efficacy on organizational commitment and ethical behavior: the role of job satisfaction. J. Theor. Appl. Manag. 16 2, 321–338. 10.20473/jmtt.v16i2.43769

[B6] Asgharian P. Panchea A. M. Ferland F. (2022). A review on the use of Mobile service robots in elderly care. Robotics 11, 127. 10.3390/robotics11060127

[B7] Bandura A. (1977). Self-efficacy: toward a unifying theory of behavioral change. Psychol. Rev. 84, 191–215. 10.1037//0033-295x.84.2.191 847061

[B8] Bandura A. (1986). Social Foundations of Thought and Action: A Social Cognitive Theory. Englewood Cliffs, NJ: Prentice-Hall.

[B9] Bandura A. (2018). Toward a psychology of human agency: pathways and reflections. Perspect. Psychol. Sci. 13 (2), 130–136. 10.1177/1745691617699280 29592657

[B10] Bandura A. (1997). “Self-efficiency,” in Encyclopedia of Human Behavior. Editor Ramachandran V. S. (New York, NY: Academic Press), 4, 71–81. 10.1037/10522-094

[B11] Bandura A. (2006). “Guide for constructing self-efficacy scales,” in Self-Efficacy Beliefs of Adolescents. Editors Pajares F. Urdan T. (Greenwich, CT: Information Age Publishing), 5, 307–337.

[B12] Bartneck C. Kulić D. Croft E. Zoghbi S. (2009). Measurement instruments for the anthropomorphism, animacy, likeability, perceived intelligence, and perceived safety of robots. Int. J. Soc. Robot. 1, 71–81. 10.1007/s12369-008-0001-3

[B13] Baxter P. Kennedy J. Senft E. Lemaignan S. Belpaeme T. (2016). “From characterising three years of HRI to methodology and reportingrecommendations,” in 2016 11th ACM/IEEE International Conference on Human-RobotInteraction (HRI). Christchurch, New Zealand. 10.1109/HRI.2016.7451777

[B14] Belpaeme T. Vogt P. van den Berghe R. Bergmann K. Göksun T. de Haas M. (2018). Guidelines for designing social robots as second language tutors. Int. J. Soc. Robot. 10 (3), 325–341. 10.1007/s12369-018-0467-6 30996752 PMC6438435

[B15] Bishop L. van Maris A. Dogramadzi S. Zook N. (2019). Social robots: the influence of human and robot characteristics on acceptance. Paladyn J. Behav. Robot. 10 (1), 346–358. 10.1515/pjbr-2019-0028

[B16] Blaurock M. Čaić M. Henkel A. P. Smedlund A. Holmlid S. (2022). A transdisciplinary review and framework of consumer interactions with embodied social robots: design, delegate, and deploy. Int. J. Consum. Stud. 46 (5), 1641–1663. 10.1111/ijcs.12808

[B17] Bostrom R. P. Heinen J. S. (1977). MIS problems and failures: a socio technical perspective. Part I: the causes. MIS Q. 1 (3), 17–32. 10.2307/248710

[B18] Braun V. Clarke V. (2006). Using thematic analysis in psychology. Qual. Res. Psychol. 3 (2), 77–101. 10.1191/1478088706qp063oa

[B19] Bražinová I. Kalenda Vávrová S. Mali J. (2024). “The introduction of social robots into the social work practice with older adults: a challenge for the education of university students in the field of gerontechnology,” in Tech Know Learn. 10.1007/s10758-024-09795-6

[B20] Bremner P. Leonards U. (2016). Iconic gestures for robot avatars, recognition and integration with speech. Front. Psychol. 7, 183. 10.3389/fpsyg.2016.00183 26925010 PMC4756113

[B21] Carpinella C. M. Wyman A. B. Perez M. A. Stroessner S. J. (2017). The robotic social attributes scale (RoSAS): development and validation. Proc. ACM/IEEE Int. Conf. Human-Robot Interact. (HRI), 254–262. 10.1145/2909824.3020208

[B22] Carradore M. (2022). People’s attitudes towards the use of robots in the social services: a multilevel analysis using Eurobarometer data. Int. J. Soc. Robot. 14 (4), 845–858. 10.1007/s12369-021-00831-4

[B23] Chen N. (2018). “Acceptance of social robots by aging sers: towards a pleasure-oriented view,” in Cross-Cultural Design. Editor Rau P. L. (Cham: Springer). 10911. 10.1007/978-3-319-92141-9_30

[B24] Cho Y. Kwon H. Y. (2025). Understanding service robot adoption and resistance from a service provider perspective. Technol. Forecast Soc. Change 210, 123885. 10.1016/j.techfore.2024.123885

[B25] Chu L. Chen H. Cheng P. Y. Ho P. Weng I. T. Yang P. L. (2019). Identifying features that enhance older adults’ acceptance of robots: a mixed methods study. Gerontology 65, 441–450. 10.1159/000494881 30844813

[B26] Darling K. (2012). Extending legal protection to social robots: the effects of anthropomorphism, empathy, and violent behavior towards robotic objects (Miami: University of Miami). 10.2139/ssrn.2044797

[B27] Davis F. D. (1989). Perceived usefulness, perceived ease of use, and user acceptance of information technology. MIS Q. 13 (3), 319–340. 10.2307/249008

[B28] Degeurin M. (2024). Can this robot help solve a guide dog shortage? Popular Sci. Available online at: https://www.popsci.com/technology/can-this-robot-help-solve-a-guide-dog-shortage/ (Accessed March 29, 2026).

[B100] Draxler F. Buschnek D. Tavast M. Hämäläinen P. Schmidt A. Kulshrestha J. (2023). Gender, age, and technology education influence the adoption and appropriation of llms. ArXiv Prepr. 10.48550/arXiv.2310.06556

[B29] Eirale A. M. Martini M. Chiaberge M. (2025). Human following and guidance by autonomous mobile robots: a comprehensive review. IEEE Access 13, 42214–42253. 10.1109/ACCESS.2025.3548134

[B30] Fasola J. Matarić M. J. (2012). “Socially assistive robot exercise coach: motivating older adults to engage in physical exercise,” in Experimental Robotics. Editors Desai J. Dudek G. Khatib O. Kumar V. (Heidelberg: Springer), 88, 463–479. 10.1007/978-3-319-00065-7_32

[B31] Feil-Seifer D. Matarić M. J. (2005). “Defining socially assistive robotics,” in Proceedings of the 9th International Conference on Rehabilitation Robotics (ICORR).

[B32] Firmino de Souza D. Sousa S. Kristjuhan-Ling K. Dunajeva O. Roosileht M. Pentel A. (2025). Trust and trustworthiness from a human-centered perspective in human–robot interaction (HRI): a systematic literature review. Electronics 14 (8), 1557. 10.3390/electronics14081557

[B33] Frieske R. Mo X. Fang Y. Nieles J. Shi B. E. (2024). Survey of design paradigms for social robots. arXiv Prepr. 10.48550/arXiv.2407.20556

[B34] García S. Strüber D. Brugali D. Berger T. Pelliccione P. (2020). “Robotics software engineering: a perspective from the service robotics domain,” in Proceedings of the 28th ACM Joint Meeting on European Software EngineeringConference and Symposium on the Foundations of Software Engineering (ESEC/FSE 2020). New York, NY: Association for Computing Machinery. 10.1145/3368089.3409743

[B35] Gasteiger N. Hellou M. Ahn H. S. (2021). Deploying social robots in museum settings: a quasi-systematic review exploring purpose and acceptability. Int. J. Adv. Robot. Syst. 18 (6), 17298814211066740. 10.1177/17298814211066740

[B36] Giger J.-C. Piçarra N. Pochwatko G. Almeida N. Almeida A. S. (2025). Intention to work with social robots: the role of perceived robot use self-efficacy, attitudes towards robots, and beliefs in human nature uniqueness. Multimodal Technol. Interact. 9 (2), 9. 10.3390/mti9020009

[B37] González-Anleo J. M. Delbello L. Martínez-Gonzálo J. M. Gómez A. (2024). Sociodemographic impact on the adoption of emerging technologies. J. Small Bus. Strategy 34 (2), 42–50. 10.53703/001c.122089

[B38] Gross H. Meyer S. Scheidig A. Eisenbach M. Müller S. Trinh T. Q. (2017). “Mobile robot companion for walking training of stroke patients in clinical post-stroke rehabilitation,” in Proceedings of the IEEE International Conference on Robotics and Automation (ICRA), 1028–1035. 10.1109/ICRA.2017.7989124

[B39] Heslin P. A. Klehe U. C. (2006). “Self-efficacy,” in Encyclopedia of Industrial/Organizational Psychology. Editor Rogelberg S. G. (Thousand Oaks, CA: Sage), 2, 705–708.

[B40] Hung L. Liu C. Woldum E. Au-Yeung A. Berndt A. Wallsworth C. (2019). The benefits of and barriers to using a social robot PARO in care settings: a scoping review. BMC Geriatr. 19, 232. 10.1186/s12877-019-1244-6 31443636 PMC6708202

[B41] Iio T. Satake S. Kanda T. Hayashi K. Ferreri F. Hagita N. (2020). Human-like guide robot that proactively explains exhibits. Int. J. Soc. Robot. 12, 549–566. 10.1007/s12369-019-00587-y

[B42] Jadad-Garcia T. Jadad A. A. (2024). The foundations of computational management: a systematic approach to task automation for the integration of artificial intelligence into existing workflows. arXiv Prepr. 10.48550/arXiv.2402.05142

[B43] Jones N. Loizides F. Jones K. (2025). Acceptance of needy socially assistive robots: a systematic review. Int. J. Soc. Robot. 17 (7), 1289–1303. 10.1007/s12369-025-01272-z

[B44] Kennedy J. Baxter P. Belpaeme T. (2017). The impact of robot tutor nonverbal social behavior on child learning. Front. ICT 4, 6. 10.3389/fict.2017.00006

[B45] Kong X. Yuan W. Ma C. (2025). Service robots, artificial intelligence awareness, self-efficacy and work engagement. Manag. Decis. 10.1108/MD-05-2024-1058

[B46] Konijn E. A. Hoorn J. F. (2020). Differential facial articulacy in robots and humans elicit different levels of responsiveness, empathy, and projected feeling. Robotics 9 (4), 92. 10.3390/robotics9040092

[B47] Kotter J. P. (1996). Leading Change. Boston: Harvard Business School Press.

[B48] Latikka R. Turja T. Oksanen A. (2019). Self-efficacy and acceptance of robots. Comput. Hum. Behav. 93, 157–163. 10.1016/j.chb.2018.12.017

[B49] Lee I. (2021). Service robots: a systematic literature review. Electronics 10 (21), 2658. 10.3390/electronics10212658

[B50] Leite I. Martinho C. Paiva A. (2013). Social robots for long-term interaction: a survey. Int. J. Soc. Robot. 5 (2), 291–308. 10.1007/s12369-013-0178-y

[B51] Leoste J. Marmor K. Heidmets M. (2024). Nonverbal behavior of service robots in social interactions: a survey on recent studies. Interact. Des. Archit. (61), 2487–2489. 10.55612/s-5002-061-006

[B52] Leoste J. Marmor K. Hollstein T. Hinkelmann H. Leoste L. B. (2024). “Enhancing university visitor satisfaction: a human-robot interaction study on the design and perception of a guide robot assistant,” in Robotics in Education (RiE 2024). Lecture Notes in Networks and Systems. Editors Balogh R. Obdržálek D. Fislake M. (Cham: Springer), 1084, 185–193. 10.1007/978-3-031-67059-6_20

[B53] Li Y. Sekino H. Sato-Shimokawara E. Yamaguchi T. (2022). The influence of robot’s expressions on self-efficacy in erroneous situations. J. Adv. Comput. Intell. Intell. Inf. 26 (4), 521–530. 10.20965/jaciii.2022.p0521

[B54] Liao S. Lin L. Chen Q. (2023). Research on the acceptance of collaborative robots for the industry 5.0 era: the mediating effect of perceived competence and the moderating effect of robot use self-efficacy. Int. J. Ind. Ergon. 95, 103455. 10.1016/j.ergon.2023.103455

[B55] Lim X. J. Chang J. Y.-S. Cheah J.-H. Lim W. M. Kraus S. Dabić M. (2024). Out of the way, human! understanding post-adoption of last-mile delivery robots. Technol. Forecast Soc. Change 201, 123242. 10.1016/j.techfore.2024.123242

[B56] Lin C. S. Kuo Y. Wang T. (2025). Trust and acceptance of AI caregiving robots: the role of ethics and self-efficacy. Comput. Hum. Behav. Artif. Hum. 3, 100115. 10.1016/j.chbah.2024.100115

[B57] Luo C. Yang C. Yuan R. Liu Q. Li P. He Y. (2024). Barriers and facilitators to technology acceptance of socially assistive robots in older adults: a qualitative study based on the capability, opportunity, and motivation behavior model (COM-B) and stakeholder perspectives. Geriatr. Nurs. 58, 162–170. 10.1016/j.gerinurse.2024.05.025 38815538

[B58] Maj K. Savicki K. Samson K. (2023). Ready or not? Examining acceptance and fears of robots in the labor market: a survey of a Polish sample. Rocz. Psychol. 26 (4), 7–24. 10.18290/rpsych2023.0019

[B59] Mandl S. Bretschneider M. Asbrock F. Meyer B. Strobel A. (2022). “The social perception of robots scale (SPRS): developing and testing a scale for successful interaction between humans and robots,” in Collaborative Networks in Digitalization and Society 5.0 (PRO-VE 2022). IFIP Advances in Information and Communication Technology. Editors Camarinha-Matos L. M. Ortiz A. Boucher X. Osório A. L. (Cham: Springer), 662, 321–334. 10.1007/978-3-031-14844-6_26

[B60] Maniscalco U. Minutolo A. Storniolo P. Esposito M. (2024). Towards a more anthropomorphic interaction with robots in museum settings: an experimental study. Robot. Auton. Syst. 171, 104561. 10.1016/j.robot.2023.104561

[B61] Mendoza S. Oliinyk S. Patiño P. Paillacho J. Hernandez D. Juan D. (2024). “Exploring the perceptions and challenges of social robot navigation: two case studies in different socio-technical contexts,” in Proceedings of the 36th Australian Conference on Human-Computer Interaction (OzCHI).

[B62] Moro C. Lin S. Nejat G. Mihailidis A. (2019). Social robots and seniors: a comparative study on the influence of dynamic social features on human–robot interaction. Int. J. Soc. Robot. 11, 5–24. 10.1007/s12369-018-0488-1

[B63] Moseley C. Mead R. (2023). Tracing the gender confidence gap in computing: a cross-national meta-analysis of gender differences in self-assessed technological ability. Comput. Educ. 198, 104735. 10.1016/j.compedu.2023.104735 36898792

[B64] Mosquera-Maturana J. S. Hernández Vega J. D. Romero C. V. (2025). “Sampling-based motion planning for guide robots considering user pose uncertainty,” in Towards Autonomous Robotic Systems (TAROS 2024) (Cham: Springer), 15051. 10.1007/978-3-031-72059-8_14

[B65] Mukherjee S. Baral M. M. Venkataiah C. Pal S. K. Nagariya R. (2021). Service robots are an option for contactless services due to the COVID-19 pandemic in the hotels. J. Inst. Eng. India Ser. A 48 (4), 445–460. 10.1007/s40622-021-00300-x

[B66] Mustafa A. Glavee-Geo R. Gronhaug K. Saber Almazrouei H. (2019). The impact of organizational structure on employee attitudes and self-efficacy. Sustainability 11 (3), 860. 10.3390/su11030860

[B67] Nomura T. Kanda T. (2021). Experiences, knowledge of functions, and social acceptance of robots: an exploratory case study focusing on Japan. AI Soc. 37 (4), 1497–1509. 10.1007/s00146-021-01196

[B68] Ogle A. Lamb D. (2019). “The role of robots, artificial intelligence, and service automation in events,” in Robots, Artificial Intelligence, and Service Automation in Travel, Tourism and Hospitality. Editors Ivanov S. Webster C. (Bingley: Emerald Publishing), 255–269. 10.1108/978-1-78756-687-320191012

[B69] Pagallo U. (2013). Robots in the cloud with privacy: a new threat to data protection. Comput. Law Secur Rev. 29 (5), 501–508. 10.1016/j.clsr.2013.07.012

[B70] Pinto A. Sousa S. Simões A. Santos J. (2022). A trust scale for human-robot interaction: translation, adaptation, and validation of a human computer trust scale. Hum. Behav. Emerg. Technol. 2022 (1), 6437441. 10.1155/2022/6437441

[B71] QViro (2024). Humanoid robots in China 2024. Available online at: https://qviro.com/blog/humanoid-robots-in-china-2024/ (Accessed March 29, 2026).

[B72] Rifinski D. Erel H. Feiner A. Hoffman G. Zukerman O. (2020). Human-human-robot interaction: robotic object’s responsive gestures improve interpersonal evaluation in human interaction. Hum. Comput. Interact. 35 (4), 1–27. 10.1080/07370024.2020.1719839

[B73] Rivero-Ortega J. D. Mosquera-Maturana J. S. Pardo-Cabrera J. Hurtado-López J. Hernández J. D. Romero-Cano V. (2023). Ring attractor bio-inspired neural network for social robot navigation. Front. Neurorobot 17, 1211570. 10.3389/fnbot.2023.1211570 37719331 PMC10501606

[B74] Robinson N. L. Hicks T.-N. Suddrey G. Kavanagh D. J. (2020). “The robot self-efficacy scale: robot self-efficacy, likability and willingness to interact increases after a robot-delivered tutorial,” in Proceedings of the IEEE International Conference on Robot and Human Interactive Communication (RO-MAN), 272–277. 10.1109/RO-MAN47096.2020.9223535

[B75] Romero-C de Vaca A. J. Meléndez-Armenta R. A. Ponce H. (2024). Using social robotics to identify educational behavior: a survey. Electronics 13 (19), 3956. 10.3390/electronics13193956

[B76] Rosenthal-von der Pütten A. Bock N. (2018). Development and validation of the self-efficacy in human-robot-interaction scale (SE-HRI). ACM Trans. Hum. Robot. Interact. 7 (3), 1–3. 10.1145/3139352

[B77] Sadangharn P. (2022). Acceptance of robots as co-workers: hotel employees’ perspective. Int. J. Eng. Bus. Manag. 14, 1–12. 10.1177/18479790221113621

[B78] Saunderson S. Nejat G. (2019). How robots influence humans: a survey of nonverbal communication in social human–robot interaction. Int. J. Soc. Robot. 11 (4), 575–608. 10.1007/s12369-019-00523-0

[B79] Savela N. Latikka R. Oksa R. Kortelainen S. Oksanen A. (2022). Affective attitudes toward robots at work: a population-wide four-wave survey study. Int. J. Soc. Robot. 14, 1379–1395. 10.1007/s12369-022-00877-y 35464870 PMC9012866

[B80] Seikkula-Leino J. Salomaa M. (2020). Entrepreneurial competencies and organisational change, assessing entrepreneurial staff competencies within higher education institutions. Sustainability 12 (18), 7323. 10.3390/su12187323

[B81] Shahzad K. Khan S. A. Iqbal A. (2024). Factors influencing the adoption of robotic technologies in academic libraries: a systematic literature review (SLR). J. Librariansh. Inf. Sci. 57, 687–704. 10.1177/09610006241231012

[B83] Spatola N. Kühnlenz B. Cheng G. (2021). Perception and evaluation in human–robot interaction: the human–robot interaction evaluation scale (HRIES), a multicomponent approach of anthropomorphism. Int. J. Soc. Robot. 13, 1517–1539. 10.1007/s12369-020-00667-4

[B84] Tachi S. Komoriya K. (1985). “Guide dog robot,” in The Robotics Research 2: Proceedings of the 2nd International Symposium of Robotics Research. (Cambridge, MA: MIT Press), 333–349. Available online at: https://tachilab.org/content/files/publication/tp/tachi1985MIT.pdf.

[B85] Takayama L. Pantofaru C. (2009). “Influences on proxemic behaviors in human-robot interaction,” in Proceedings of the IEEE/RSJ International Conference on Intelligent Robots and Systems (IROS), 5495–5502. 10.1109/IROS.2009.5354145

[B86] Tan N. Mohan R. E. Watanabe A. (2016). Toward a framework for robot-inclusive environments. Autom. Constr. 69, 68–78. 10.1016/j.autcon.2016.06.001

[B87] Tanase G. C. (2023). Social robots in organizational contexts: the role of culture and future research need. J. Res. Dev. Stud. 14 (3), 25–32. Available online at: https://ideas.repec.org/a/rdc/journl/v14y2023i3p25-32.html. (Accessed March 29, 2026)

[B88] Thrun S. Bennewitz M. Burgard W. Cremers A. B. Dellaert F. Fox D. (2000). “Minerva: a second-generation museum tour-guide robot,” in Proceedings of the IEEE International Conference on Robotics and Automation (ICRA), 1999–2005. 10.1109/ROBOT.1999.770401

[B89] Turja T. Rantanen T. Oksanen A. (2019). Robot use self-efficacy in healthcare work (RUSH): development and validation of a new measure. AI Soc. 34, 137–143. 10.1007/s00146-017-0751-2

[B90] Turja T. Taipale S. Kaakinen M. Oksanen A. (2020a). Care workers’ readiness for robotization: identifying psychological and socio-demographic determinants. Int. J. Soc. Robot. 12, 79–90. 10.1007/s12369-019-00544-9

[B91] Turja T. Aaltonen I. Taipale S. Oksanen A. (2020b). Robot acceptance model for care (RAM-care): a principled approach to the intention to use care robots. Inf. Manag. 57, 103220. 10.1016/j.im.2019.103220

[B92] Venkatesh V. Morris M. G. Davis G. B. Davis F. D. (2003). User acceptance of information technology: toward a unified view. MIS Q. 27 (3), 425–478. 10.2307/30036540

[B93] Vincent J. Taipale S. Sapio B. Lugano G. Fortunati L. (2015). Social Robots from a Human Perspective (Cham: Springer).

[B94] Vishwakarma L. P. Singh R. K. Mishra R. Demirkol D. Daim T. (2024). The adoption of social robots in service operations: a comprehensive review. Technol. Soc. 76, 102441. 10.1016/j.techsoc.2023.102441

[B95] Wirtz J. Patterson P. G. Kunz W. H. Gruber T. Lu V. N. Paluch S. (2018). Brave new world: service robots in the frontline. J. Serv. Manag. 29 (5), 907–931. 10.1108/JOSM-04-2018-0119

[B96] Wu X. Li X. Zhang C. Luo X. R. Du W. (2025). How artificial intelligence affects employee job insecurity: the roles of knowledge sharing and work innovation. J. Bus. Res. 144, 151–161. 10.1016/j.jbusres.2022.12.030

[B97] Yi E. Park D.-H. (2024). Potential user segmentation based on expectations of social robots using Q-methodology. IEEE Access 12, 100295–100315. 10.1109/ACCESS.2024.3430864

[B98] Yoshimitsu K. Shiigi Y. Kobayashi E. Kuwana Y. Kusuda K. Hoshide K. (2024). Exploring the potential of service robots in guiding patients and assisting mobility in medical facilities. Adv. Robot. 38 (24), 1758–1769. 10.1080/01691864.2024.2438653

[B99] Zeller F. Dwyer L. D. (2022). Systems of collaboration: challenges and solutions for interdisciplinary research in AI and social robotics. Discov. Artif. Intell. 2, 12. 10.1007/s44163-022-00027-3

