# Supplementary material for: Guide robots’ acceptance in organizations: user self-efficacy and robots’ organizational value
Source: Front Robot AI. 2026 Jun 3;13:1842941. doi: 10.3389/frobt.2026.1842941 (PMC13272404; doi:10.3389/frobt.2026.1842941)
Supplement: Supplementary file 1 [file Supplementaryfile1.docx]

# Appendix 1

*The GRUSES Data Collection Instrument*

Hello!

Please rate your experience of interacting with the robot today! Answer the following questions by clicking on the appropriate answer option. If you don't want to or can't answer a question or statement, just skip it. Your answers will remain anonymous, it will take 6-7 minutes to answer. Please confirm with your signature and date that you agree to the use of the data collected from you through this questionnaire for the preparation of research articles and that you are participating voluntarily.

Date: ...................... Name: .............................. Signature: ..............................

Thank you, IT Didactics Research Group, TalTech IT College.

**Thinking about your experience today, how do you rate your interaction/communication with the robot?**

|  | 1 | 2 | 3 | 4 | 5 | 6 | 7 |
| --- | --- | --- | --- | --- | --- | --- | --- |
| **Difficulty.** Communicating with the robot seem to you... | Very difficult | Difficult | Rather Difficult | So-so | Rather Easy | Easy | Very Easy |
| **Understandability.** How did you understand the robot’s messages/conversations? | Very Badly | Badly | Rather Badly | So-so | Rather Well | Well | Very Well |
| **Location.** Was the location of the robot during communication...? | Very Unsuitable | Unsuitable | Rather Unsuitable | So-so | Rather Suitable | Suitable | Very Suitable |
| **Security**. Do you consider interacting with the robot... | Very Unsecure | Unsecure | Rather Unsecure | So-so | Rather Secure | Secure | Very Secure |
| **Reliability**. Do you consider the robot as a communication partner for you... | Very Unreliable | Unreliable | Rather Unreliable | So-so | Rather Reliable | Reliable | Very Reliable |
| **Pleasantness**. Do you consider the robot as a communication partner for you... | Very Unpleasant | Unpleasant | Rather Unpleasant | So-so | Rather Pleasant | Pleasant | Very Pleasant |
| **Self-confidence.** How confident or uncertain did you feel during your interaction with a robot? | Very uncertain | Uncertain | Rather uncertain | So-so | Rather confident | Confident | Very confident |

**Think about the tasks you solved in cooperation with the robot today. How would you assess the completion of these tasks (such as moving from one room to another, etc.) together with the robot?**

| **Effectiveness**. Do you consider that collaborating with the robot helped to complete the tasks better or worse? | Significantly worse | Worse | Rather worse | So-so | Rather better | Better | Significantly better |
| --- | --- | --- | --- | --- | --- | --- | --- |
| **Comfort.** How convenient was it for you to solve today's tasks in cooperation with the robot? | Very uncomfortable | Uncomfortable | Rather uncomfortable | So-so | Rather comfortable | Comfortable | Very comfortable |
| **Speed.** Do you consider working with the robot make solving tasks faster or slower for you? | Significantly slower | Slower | Rather slower | So-so | Rather faster | Faster | Significantly faster |
| **Suitability.** Was the cooperation with a robot just for solving such tasks (like today's)... | Very inappropriate | Inappropriate | Rather inappropriate | So-so | Rather suitable | Suitable | Very suitable |
| **Naturality.** How natural was solving tasks with a robot for you? | Very unnatural | Unnatural | Rather unnatural | So-so | Rather natural | Natural | Very natural |

**While you were working with the robot, other people were also present in the room. What was their attitude towards the interaction between you and the robot and acting together?**

| **Attention.** To what extent was attention paid to your and the robot's activities? | No attention was paid at all | In general, no attention was paid | There was little attention | So-so | Some attention was paid | attention was paid | There was a lot of attention |
| --- | --- | --- | --- | --- | --- | --- | --- |
| **Intervention.** To what extent did bystanders interfere in your and the robot's activities? | Didn't interfere at all | Didn't intervene | Rather did not intervene | So-so | Rather intervened | Intervened | There was a lot of interference |
| **Acting in front of others.** Was working with a robot under the eyes of other people for you... | Very unpleasant | Unpleasant | Rather unpleasant | So-so | Rather pleasant | Pleasant | Very pleasant |

**And finally!**

**To what extent has your previous work or study included communication or cooperation with a robot?** 1) not at all, 2) rarely, 3) so-so, 4) often, 5) constantly

**If in the future a robot could touch you (while acting together or solving tasks), would it be for you:** 1) very unpleasant, 2) unpleasant, 3) so-so, 4) pleasant, 5) very pleasant

**Did you perceive today's robot more as a machine or as a person?** 1) definitely as a machine, 2) rather as a machine, 3) so-so, 4) rather as a human, 5) definitely as a human

You are: female male Your age is between: up to 30 years, 31-50 years, 51 and more years

**THANK YOU!**

# Appendix 2

Table 2. *Descriptive statistics of the GRUSES scale items*

| Variables | Descriptive Statistics | | | | |
| --- | --- | --- | --- | --- | --- |
|  | Valid N | Mean | Min | Max | Std.Dev. |
| **Difficulty.** Communicating with the robot seem to you... | 226 | 5.74 | 2.00 | 7.00 | 1.07 |
| **Understandability.** How did you understand the robot’s messages/conversations? | 226 | 5.77 | 2.00 | 7.00 | 1.10 |
| **Location.** Was the location of the robot during communication...? | 226 | 5.53 | 2.00 | 7.00 | 1.11 |
| **Security**. Do you consider interacting with the robot... | 226 | 5.56 | 2.00 | 7.00 | 1.04 |
| **Reliability**. Do you consider the robot as a communication partner for you... | 226 | 5.29 | 2.00 | 7.00 | 1.13 |
| **Pleasantness**. Do you consider the robot as a communication partner for you... | 226 | 5.60 | 2.00 | 7.00 | 1.17 |
| **Self-confidence.** How confident or uncertain did you feel during your interaction with a robot? | 226 | 5.26 | 2.00 | 7.00 | 1.38 |
| **Effectiveness**. Do you consider that collaborating with the robot helped to complete the tasks better or worse? | 221 | 5.29 | 1.00 | 7.00 | 1.09 |
| **Comfort.** How convenient was it for you to solve today's tasks in cooperation with the robot? | 220 | 5.44 | 2.00 | 7.00 | 1.14 |
| **Speed.** Do you consider working with the robot make solving tasks faster or slower for you? | 220 | 4.68 | 1.00 | 7.00 | 1.26 |
| **Suitability.** Was the cooperation with a robot just for solving such tasks (like today's)... | 220 | 5.78 | 3.00 | 7.00 | 0.89 |
| **Naturality.** How natural was solving tasks with a robot for you? | 220 | 4.94 | 2.000 | 7.00 | 1.31 |

# Appendix 3

Table 3. *Reliability of the GRUSES scale*

| Variable | Summary for scale: Mean=64.80 Std.Dv. =9.62873 Valid N:217 Cronbach **alpha: 0.904** Standardized alpha: 0.906 Average inter-item corr.: 0.451 | | | | |
| --- | --- | --- | --- | --- | --- |
|  | Mean if  Deleted | Var. If  deleted | StDv. If  deleted | Itm-Totl  Correl | Alpha if  Deleted |
| **Difficulty.** Communicating with the robot seem to you... | 59.06 | 78.14 | 8.839 | 0.684 | 0.895 |
| **Understandability.** How did you understand the robot’s messages/conversations? | 59.05 | 80.09 | 8.949 | 0.557 | 0.900 |
| **Location.** Was the location of the robot during communication...? | 59.29 | 79.44 | 8.913 | 0.578 | 0.899 |
| **Security**. Do you consider interacting with the robot... | 59.24 | 78.39 | 8.854 | 0.702 | 0.894 |
| **Reliability**. Do you consider the robot as a communication partner for you... | 59.52 | 78.32 | 8.850 | 0.637 | 0.897 |
| **Pleasantness**. Do you consider the robot as a communication partner for you... | 59.23 | 77.02 | 8.776 | 0.680 | 0.895 |
| **Self-confidence.** How confident or uncertain did you feel during your interaction with a robot? | 59.55 | 74.63 | 8.639 | 0.662 | 0.896 |
| **Effectiveness**. Do you consider that collaborating with the robot helped to complete the tasks better or worse? | 59.52 | 77.93 | 8.828 | 0.687 | 0.894 |
| **Comfort.** How convenient was it for you to solve today's tasks in cooperation with the robot? | 59.38 | 76.55 | 8.749 | 0.728 | 0.892 |
| **Speed.** Do you consider working with the robot make solving tasks faster or slower for you? | 60.13 | 79.06 | 8.891 | 0.524 | 0.903 |
| **Suitability.** Was the cooperation with a robot just for solving such tasks (like today's)... | 59.04 | 82.58 | 9.088 | 0.556 | 0.901 |
| **Naturality.** How natural was solving tasks with a robot for you? | 59.86 | 76.45 | 8.744 | 0.617 | 0.898 |

# Appendix 4

Table 4. *Factor analysis of the GRUSES scale items*

| Variable | Factor Loadings (Unrotated) Extraction  Principal components (Marked loadings are >.600000) |
| --- | --- |
|  | Factor |
| **Difficulty.** Communicating with the robot seem to you... | 0.74 |
| **Understandability.** How did you understand the robot’s messages/conversations? | 0.63 |
| **Location.** Was the location of the robot during communication...? | 0.65 |
| **Security**. Do you consider interacting with the robot... | 0.76 |
| **Reliability**. Do you consider the robot as a communication partner for you... | 0.71 |
| **Pleasantness**. Do you consider the robot as a communication partner for you... | 0.75 |
| **Self-confidence.** How confident or uncertain did you feel during your interaction with a robot? | 0.73 |
| **Effectiveness**. Do you consider that collaborating with the robot helped to complete the tasks better or worse? | 0.75 |
| **Comfort.** How convenient was it for you to solve today's tasks in cooperation with the robot? | 0.78 |
| **Speed.** Do you consider working with the robot make solving tasks faster or slower for you? | 0.60 |
| **Suitability.** Was the cooperation with a robot just for solving such tasks (like today's)... | 0.62 |
| **Naturality.** How natural was solving tasks with a robot for you? | 0.68 |
